# A cross-sectional study of cosmetic surgery tendency and its psychological and media-related factors among medical students in 2024–2025

**DOI:** 10.1371/journal.pone.0360409

**Published:** 2026-10-30

**Authors:** Parviz Shahmirzalou, Vahid Sabri, Hassan Ebrahimpour Sadagheyani

**Affiliations:** 1 Department of Public Health, Khoy University of Medical Sciences, Khoy, Iran; 2 Clinical Research Development Unit, Ayatollah Khoei Educational & Treatment Center, Khoy University of Medical Sciences, Khoy, Iran; 3 Department of Basic Medical Sciences, Khoy University of Medical Sciences, Khoy, Iran; 4 Department of Health Information Technology, Khoy University of Medical Sciences, Khoy, Iran; 5 Department of Health Information Technology, Neyshabur University of Medical Sciences, Neyshabur, Iran; Muhammad Nawaz Sharif University of Engineering and Technology, PAKISTAN

## Abstract

**Background:**

Over 15 million plastic surgeries and 19 million non-plastic aesthetic procedures were carried out globally between 2020 and 2023, representing a substantial growth of more than 40%, according to the International Society of Aesthetic Plastic Surgery (ISAPS). The purpose of this study was to ascertain the degree of propensity for cosmetic surgery among Khoy University of Medical Sciences students and investigate its associations with demographic features, the frequency of watching content related to beauty (per day), perceived body image, and self-esteem.

**Materials and methods:**

This analytical cross-sectional study included 196 students from Khoy University of Medical Sciences, selected via convenience sampling for the 2024–2025 academic year. Valid and reliable questionnaires were used to gather data: 69 items on perceived body image, 10 items on self-esteem, and 24 items on tendency toward cosmetic surgery. Multiple linear regression, ANOVA, and independent t-tests were used to analyze the data in SPSS version 26.

**Results:**

The mean age of participants was 23 years. The majority were female (86%), single (94%), enrolled in health and basic sciences (43%), at the bachelor level (75%), and reported a monthly income of more than 300 million Iranian Rials (52%). No statistically significant differences were observed between demographic features and mean tendency toward cosmetic surgery (P > 0.05). However, a high frequency of watching beauty‑related content per day (β = 10.06, P < 0.001) and low self‑esteem (β = −1.25, P < 0.001) were significantly associated with a greater tendency toward cosmetic surgery.

**Conclusion:**

Students’ tendency toward cosmetic surgery is influenced by their self-esteem, and even the frequency of exposure to beauty-related content.

## Introduction

In recent years, elective cosmetic surgeries have increased substantially worldwide, including in Iran. Cosmetic surgeries are performed to alter appearance and improve aesthetics rather than to treat medical conditions. Common procedures include rhinoplasty, liposuction, botulinum toxin injection, and hair transplantation [1]. Global statistics show a sharp rise in the number of cosmetic procedures in recent years. According to the International Society of Aesthetic Plastic Surgery (ISAPS), between 2020 and 2023, more than 15.8 million plastic surgeries and 19.1 million non‑plastic procedures were performed globally, representing an increase of over 40% [1].

This rapid increase has been particularly evident in developing countries, where economic growth and greater emphasis on appearance are influential factors. The United States, for example, performed over 6.1 million cosmetic procedures in 2023, including rhinoplasty, liposuction, and breast surgery. In Europe, countries such as Germany, Spain, and Italy report high numbers of cosmetic procedures; Germany alone reported approximately 500,000 procedures in 2021 [1].

Asia has also experienced rapid growth: in South Korea, more than 13.5 per 1,000 people underwent at least one cosmetic surgery in 2011. Iran also exhibits a high rate of cosmetic surgeries, especially rhinoplasty among women; the Iranian Association of Plastic Surgeons reported over 150,000 rhinoplasties in 2021, with cultural factors, increased access to services, and social media influence cited as drivers [2].

A notable issue is the tendency of some individuals to undergo multiple cosmetic procedures (‘cosmetic surgery addiction’), which may stem from persistent dissatisfaction or internalization of unrealistic beauty standards and can lead to psychological and physical complications, including body dysmorphic disorder [3,4].University students are a vulnerable group due to academic pressures, social comparison, identity formation, and heightened concerns about appearance. Medical students are particularly interesting in this topic, because despite their greater knowledge of anatomy and procedural risks, they may still be susceptible to pressures for aesthetic perfection in academic and clinical environments [5,6].

Psychological variables such as perceived body image and self‑esteem, and environmental factors such as social media, are key factors of interest in cosmetic surgery [7]. Perceived body image—the individual’s perception, thoughts, and feelings about their body—is highly associated with interest in aesthetic procedures [8]. Low self‑esteem has also been associated with a higher inclination toward cosmetic surgery in several studies [9]. Social media platforms (Instagram, Telegram, TikTok) act as amplifiers of idealized beauty standards by presenting edited and filtered images, promoting upward social comparison, and reinforcing body dissatisfaction and reduced self‑esteem [10].

Recent studies have either explored body image, self-esteem, or media exposure separately or focused mostly on women seeking cosmetic procedures, adolescents, and the general population [7,10,11]. Thus, there is a lack of evidence concerning the joint effects of these psychological and media-related variables on cosmetic surgery susceptibility in medical students who are more health literate and knowledgeable about the risks involved in the process, but at the same time, are faced with social pressures associated with appearance [5,6]. In addition, recent systematic reviews stress the need for multivariable methods that would allow the assessment of the roles of psychological and environmental factors without considering each of them independently [7,12]. Furthermore, while self-esteem has always been considered a risk factor for cosmetic surgery [2,3,9], recent findings suggest that some people with high self-esteem seek cosmetic enhancements as a means of self-enhancement, indicating the controversy in this connection [13]. Lastly, due to the significant cultural differences in sociocultural standards of beauty, results obtained in Western cultures could not easily be applied to Middle Eastern countries like Iran, which have a high rate of cosmetic surgeries [2,7,11]. Therefore, the current study analyzed demographic characteristics, exposure to beauty-related media, body image, and self-esteem within one multivariable regression model in order to identify their independent and relative effects on cosmetic surgery susceptibility in Iranian medical students. Unlike previous studies that primarily investigated isolated psychological constructs, the present study aimed to provide an integrated evaluation of psychological and media-related determinants while identifying their relative importance within a culturally specific population of Iranian medical students [7,11,14].

Given the existing knowledge gap concerning the interaction of demographic, psychological, and media‑related factors in a specific cultural context, such as Khoy County, this study evaluated these associations in students of Khoy University of Medical Sciences and tested the following hypotheses:

Hypothesis 1: Controlling for demographic characteristics, frequent viewing of beauty‑related content increases students’ tendency toward cosmetic surgery.
Hypothesis 2: Controlling for demographic characteristics, high perceived body image decreases students’ tendency toward cosmetic surgery.
Hypothesis 3: Controlling for demographic characteristics, low self‑esteem is related to students’ tendency toward cosmetic surgery.

## Materials and methods

Study design and participants: This analytical cross‑sectional study was conducted in 2025. The study population included students of Khoy University of Medical Sciences across various fields and educational levels. To compute the sample size for the study, Kasmaei et al. used. This study assessed the relationship between the tendency to undergo cosmetic surgery and body image using logistic regression in 314 students from Guilan University of Medical Science, Guilan, Iran, who ranged from 18 to 55 years old. The authors have reported body-satisfaction (β=5.49 (95% CI:2.46−12.25, OR=3.72, p=0.01)) and body-image (β=2.28(1.32−3.94), OR=2.91), and attitudes (β=3.25(95%CI:1.46−7.20), OR=2.88, p = 0.04) were factors in the tendency toward cosmetic surgery. Using parameters from Kasmaei et al. [11] (body image odds ratio = 2.91) and assuming α = 0.05, power = 0.80, and finite population size (N = 500), the minimum sample size was calculated to be 160. Convenience (non‑probability) sampling was used. The study questionnaire was uploaded to the Porsline platform [15] and participants were invited via related social media to participate. The collection of study data started on 21/4/2025 and continued until 21/6/2025. Exactly 220 students (response rate = 89%) viewed the study questionnaire, and 196 completed it in full and were included in the analysis. Linear Regression, as the main statistical method to test the hypothesis, has 9 associated factors, and the study used approximately 22 participants for every one.

### Measures

The instrument comprised four sections: demographic characteristics, the tendency toward cosmetic surgery questionnaire (24 items), a multidimensional perceived body image questionnaire (69 items), and the Rosenberg Self‑Esteem Scale (10 items).

Perceived body image: The body image questionnaire includes 69 items scored on a 5‑point Likert scale from ‘strongly disagree’ (1) to ‘strongly agree’ (5); higher scores indicate greater body satisfaction [16]. The reported Cronbach’s alpha in prior research studies ranged from 0.77 to 0.91 for men and from 0.73 to 0.89 for women [8,17,18].

**Self‑esteem**: The Rosenberg Self‑Esteem Scale (10 items) was used; items are scored as ‘agree’ (+1) or ‘disagree’ (−1) for 1–5 questions and ‘agree’ (−1) or ‘disagree’ (+1) for 6–10 questions, with total scores ranging from −10 (Very Low self-esteem) to +10 (very high self-esteem) and a cutoff of zero [19]. Reported reliability coefficients are satisfactory (e.g., Cronbach’s alpha ≈0.84) [20–22].

**Tendency toward cosmetic surgery questionnaire**: This 24‑item scale uses a 5‑point Likert scale from ‘strongly agree’ (1) to ‘strongly disagree’ (5). Total scores range from 24 to 120, and the median score is equal to 72 ((24+120)/2=72). Students with scores more than 72 are classified as susceptible to cosmetic surgery. The questionnaire’s validity and reliability were previously reported by Etemadi Far and Amani (validity = 0.76; reliability = 0.79) [23,24].

**Ethical approval**: The study protocol was approved by the Ethics Committee of Khoy University of Medical Sciences (IR.KHOY.REC.1404.007). Written informed consent was obtained from the students. For this purpose, the study’s objectives and statistical population were described on the first page of the questionnaire. The students were also informed that their participation in the study was voluntary and that no personal information about the students would be published. No identifying information (e.g., IP addresses, names) was collected. Written consent included agreement for anonymized data publication in accordance with journal policy.

**Statistical analysis**: Mean comparisons across demographic characteristics used independent t‑tests (or Mann-Whitney test under non-parametric conditions) and one‑way ANOVA (or Kruskal-Wallis test under non-parametric conditions) at α = 0.05. Following questionnaire guidelines, participants scoring above 72, were classified as ‘susceptible’; associations between this binary status (susceptible or non-susceptible) and demographic variables were assessed with chi‑square tests. Multiple linear regression was used to evaluate relationships between the tendency to undergo cosmetic surgery, as a continuous dependent variable, and demographic features, frequency of watching beauty-related content, perceived body image, and self-esteem as explanatory variables, with checks for multicollinearity (using Variance Inflation Factor(VIF)), independence of residuals (using Durbin–Watson test), and homoscedasticity by residuals plot. All associated factors were entered simultaneously using the Enter method. Demographic features were included as covariates to adjust for potential confounding effects. All of the study variables were collected using a self-report questionnaire at a single time period. Then, to assess the possibility of common method variance (CMV), Harman’s single-factor test was used. To accomplish this, all items from all study questionnaires were included in an unrotated exploratory factor analysis. The CMV less than 50% was considered unlikely [14]. Analyses were performed using SPSS version 26.

## Results

In this study, 220 students at Khoy University of Medical Sciences were asked to complete the questionnaire, and 196 of them completed it. The mean (SD) age, tendency toward cosmetic surgery, perceived body image, and self‑esteem were 23.16 (4.87) years, 62.58 (15.11), 229.99 (20.94), and 5.72 (4.31), respectively. Half of the students were 22 years old. The medians for the tendency towards cosmetic surgery, body image, and self-esteem of the students were 60.5, 230, and 6, respectively. Also, the lowest (highest) scores on the mentioned scales were 28 (106), 168 (295), and −10 (10), respectively.

Most participants were female (86%), single (94%), enrolled in health and basic sciences fields (43%), and at the bachelor’s (BSc) level (77%). The most common reported monthly income was more than 300 million Iranian Rials (52%). Sixty‑one percent of students reported that they ‘rarely’ watched beauty‑related content. Thirty students (15%) had a history of cosmetic surgery: 23 (80%) rhinoplasty, two (7%) lip filler (augmentation), two LASIK eye procedures, one temporal lift, and one jaw‑angle contouring procedure (Table 1).

**Table 1:** The frequency (%) of the students’ features, along with the mean ± standard deviation of tendency to cosmetic surgery, body image, and self-esteem of students according to the mentioned characteristics.

Feature	Subgroups	N (%)	Mean ± SD		
			Tendency to Cosmetic Surgery	Body-Image	Self-Esteem
Gender	Female	169(86.22)	62.95 ± 14.96	230.59 ± 19.42	5.85 ± 4.35
	Male	27(13.77)	60.26 ± 16.13	226.26 ± 28.88	4.96 ± 4.01
Marital Status	Single	185(94.38)	62.65 ± 15.15	230.10 ± 20.58	5.75 ± 4.29
	Married	11(5.61)	61.36 ± 15.23	228.09 ± 27.37	5.27 ± 4.84
Field of study^*^	S1	84(42.86)	62.31 ± 15.49	232.42 ± 20.86	5.10 ± 4.59
	S2	47(23.98)	66.66 ± 14.88	225.53 ± 21.38	5.62 ± 4.08
	S3	44(22.45)	60.91 ± 14.79	230.32 ± 20.64	6.45 ± 4.27
	S4	21(10.71)	58.05 ± 13.58	229.57 ± 20.69	6.95 ± 3.44
Level of education	BSc	150(76.5)	62.99 ± 14.56	230.42 ± 20.49	5.77 ± 4.33
	MSc & PhD	46(23.5)	61.26 ± 16.89	228.59 ± 22.51	5.57 ± 4.30
Family Income (million Rials)	<300	95(48.5)	63.02 ± 14.87	231.76 ± 20.47	6.23 ± 3.90
	>300	101(51.5)	62.17 ± 15.41	228.33 ± 21.33	5.25 ± 4.63
Cosmetic surgery history	No	166(84.69)	62.23 ± 15.33	231.02 ± 20.88	5.83 ± 4.36
	Yes	30(15.31)	64.50 ± 13.97	224.30 ± 20.68	5.13 ± 4.02
The number of daily watching beauty contents	Rarely	120(61.2)	58.39 ± 12.15	229.49 ± 18.66	6.05 ± 4.08
	Usually	76(38.8)	69.20 ± 16.95	230.77 ± 24.21	5.21 ± 4.63

* S1: Health & Basic Sciences, S2: Nursing & Midwifery, S3: Medicine, Pharmacy and Dentistry, S4: Emergency Medicine, Operating Room, and Anesthesia, BSc: Bachelor of Sciences, MSc: Master of Sciences, PhD: Philosopher of Doctor.

The internal consistency (Cronbach’s alpha) of the cosmetic surgery tendency, body image, and self-esteem questionnaires was 0.86, 0.82, and 0.78, respectively. Figs 1–3 present histograms of students’ tendency towards cosmetic surgery, body image, and self-esteem with skewness and kurtosis values and a normal distribution line. Regarding gender distribution, the data concerning the tendency towards cosmetic surgery (skewness = 0.49, kurtosis = 0.05) (Fig 1), body image (skewness = 0.07, kurtosis = 0.58) (Fig 2), and self-esteem (skewness = −5.85, kurtosis = 1.93) (Fig 3) do not exhibit significant deviations from normality, except for self-esteem. To assess CMV, Harman’s Single-Factor Test is used. Harman’s single-factor test indicated that the first unrotated factor explained 17.41% of the total variance, suggesting that common method variance was unlikely to substantially influence the observed associations.

**Fig 1:**
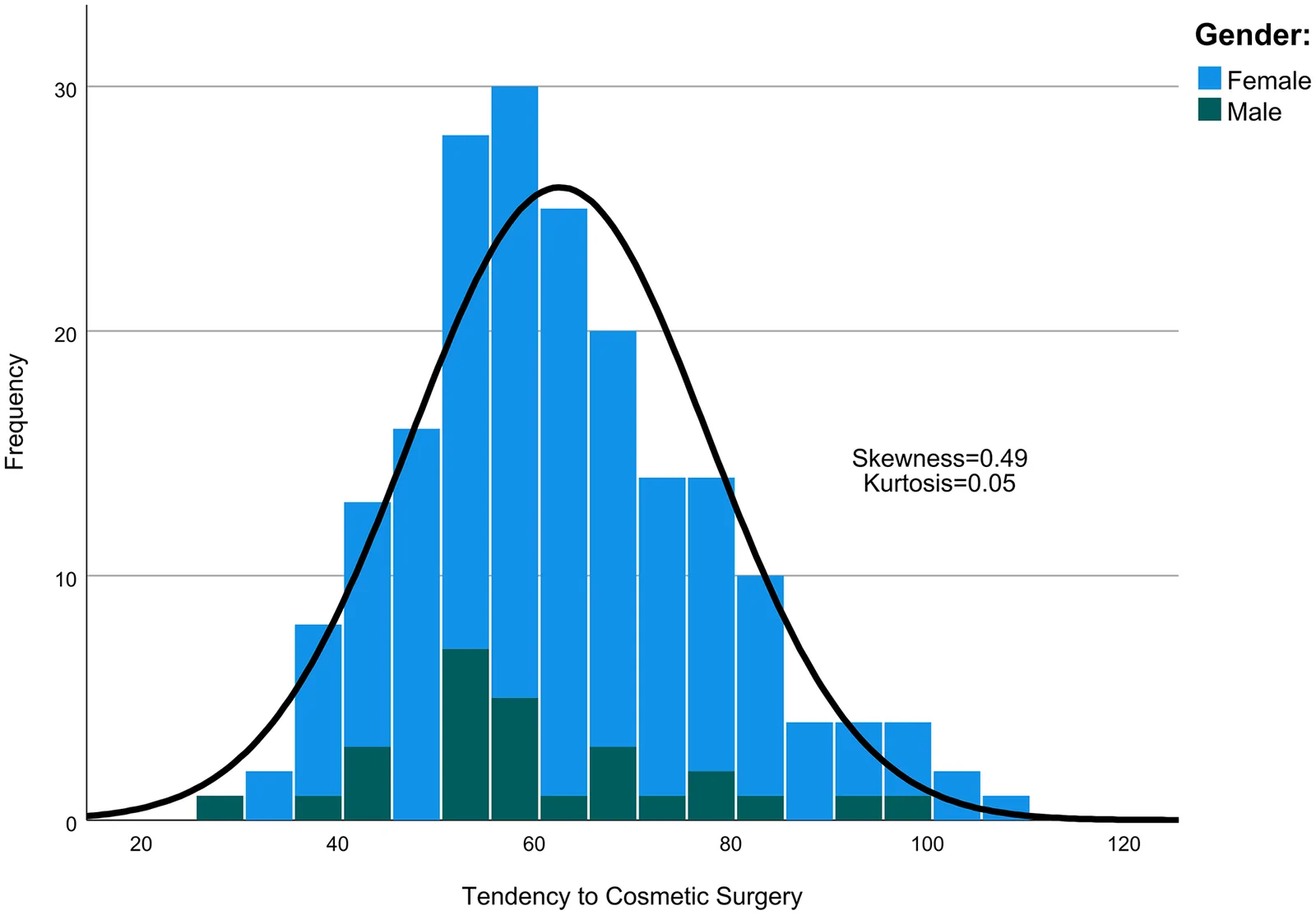
Histogram plot of students’ tendency towards cosmetic surgery by gender, along with the normal line, skewness, and kurtosis indices.

**Fig 2:**
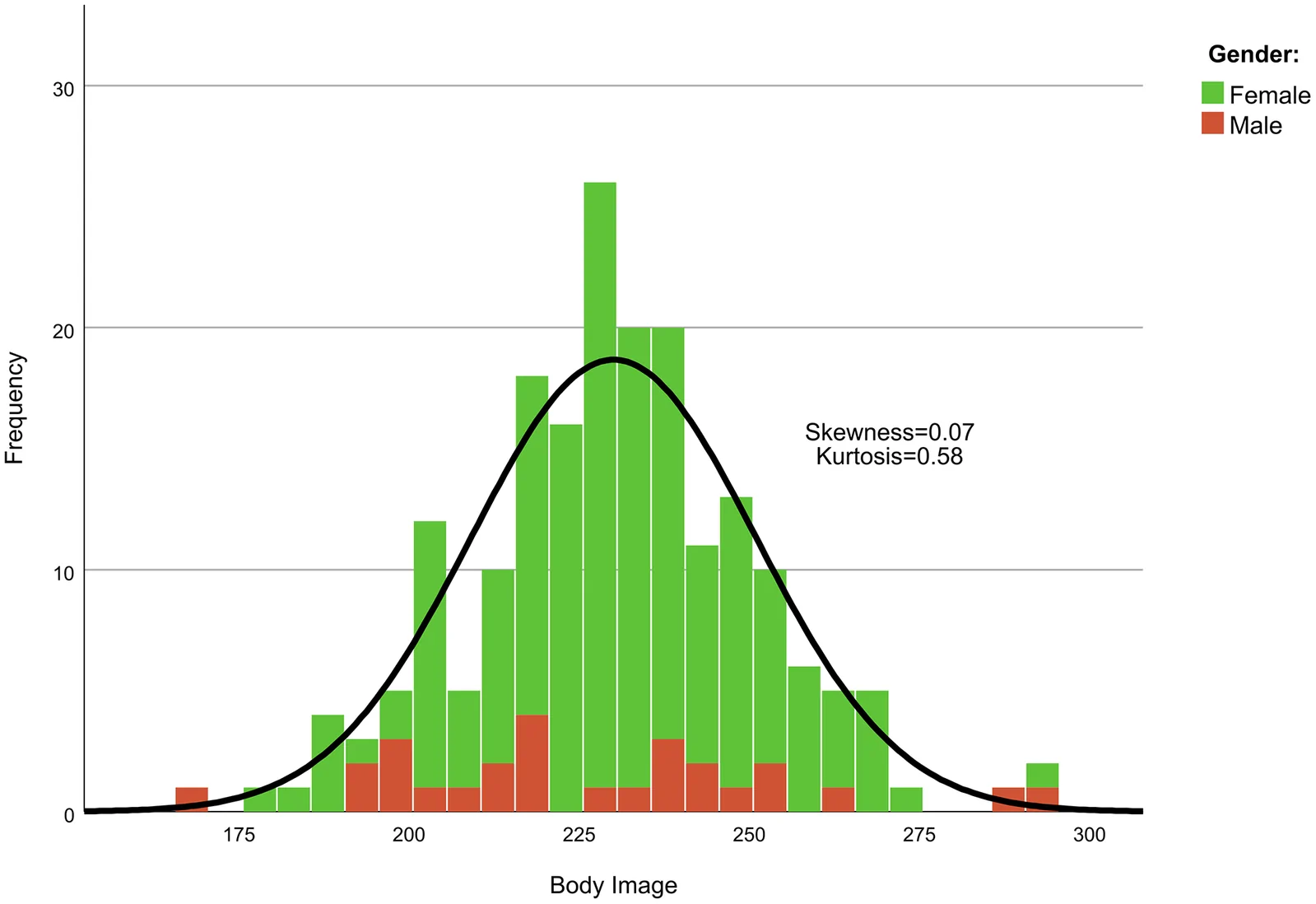
A histogram plot of students’ body image by gender, along with the normal line, skewness, and kurtosis indices.

**Fig 3:**
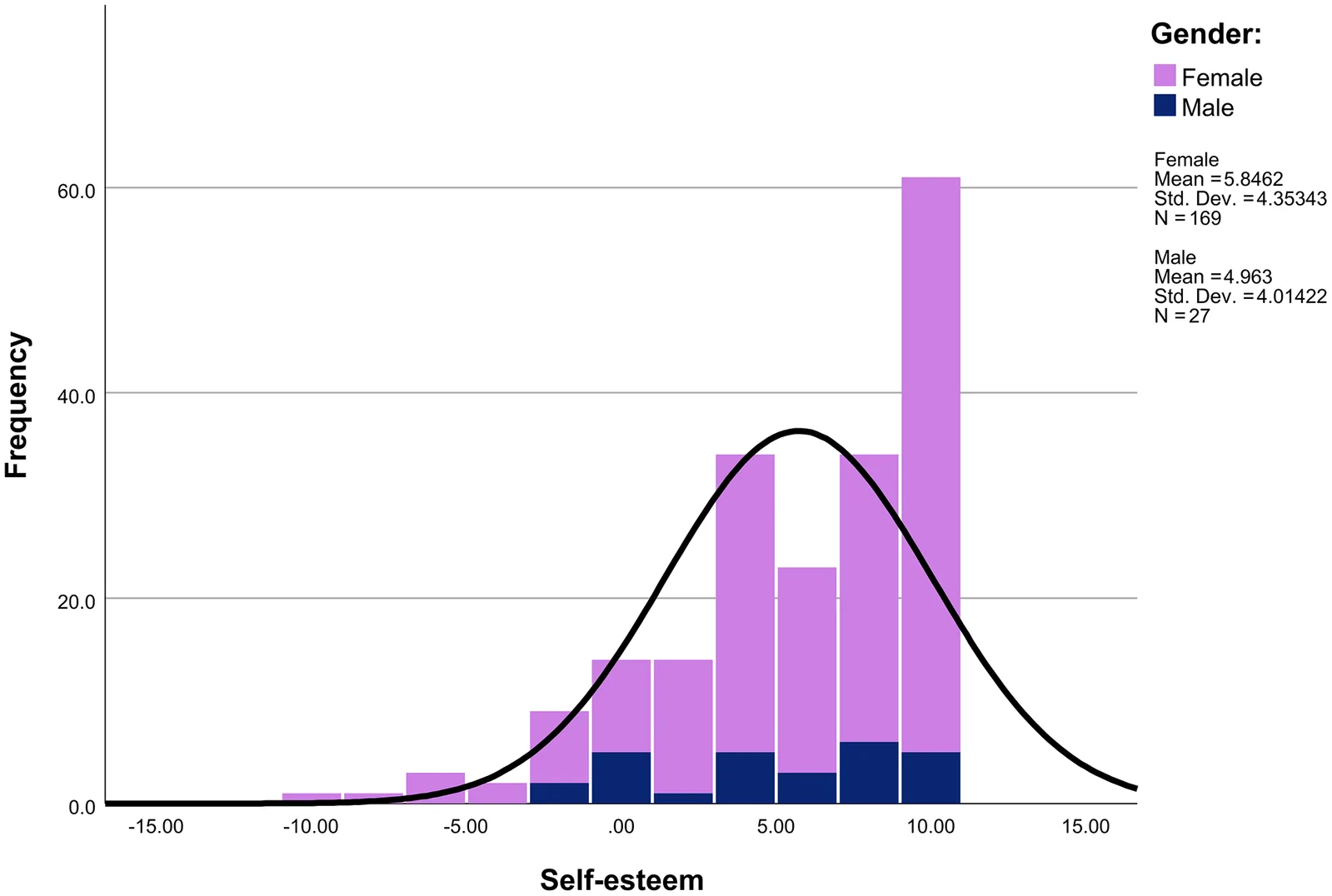
A histogram plot of students’ self-esteem by gender, along with the normal line and the skewness and kurtosis indices.

Table 2 examines the assumption that the mean of the tendency towards cosmetic surgery is equal across demographic characteristics (p > 0.05), cosmetic surgery history (p = 0.451), and the number of daily watching beauty content (significant, p < 0.001). The relationship between demographic characteristics and the tendency towards cosmetic surgery (susceptible and non-susceptible) was also examined in this table. There were no significant differences across demographic subgroups (P > 0.05). However, the frequency of watching beauty‑related content (per day) was significantly associated with mean tendency scores (P < 0.001); students who reported ‘rarely’ viewing beauty content had lower mean tendency scores (58.39 ± 12.15) compared to other categories.

**Table 2:** Investigating the assumption of equality of means of tendency towards cosmetic surgery among students with different demographic characteristics, as well as their relationship with cosmetic surgery Classification (susceptible and non-susceptible), using the Chi-square test.

Feature	Tendency to cosmetic surgery as	
	Score	Classified (susceptible^**^ and non-susceptible)
Gender	t=0.86, P=0.391	$X^{\text{2}}=\text{0}.\text{7}\text{0},\;P=\text{0}.\text{4}\text{8}\text{0}$
Marital status	t=0.27, P=0.784	$X^{\text{2}}=\text{0}.\text{8}\text{0},\;P=\text{0}.\text{4}\text{7}\text{2}$
Field of study	F = 1.90, P = 0.117	$X^{\text{2}}=\text{1}\text{0}.\text{5}\text{2},\;P=\text{0}.\text{6}\text{6}\text{2}$
Level of education	Z^*^ = −0.87, P = 0.381	$X^{\text{2}}=\text{0}.\text{0}\text{4},\;P=\text{0}.\text{8}\text{4}\text{7}$
Family Income	Z^*^ = −0.44, P = 0.662	$X^{\text{2}}=\text{0}.\text{1}\text{7},\;P=\text{0}.\text{7}\text{4}\text{2}$
Cosmetic surgery history	t=-0.76, P=0.451	$X^{\text{2}}=\text{0}.\text{4}\text{7},\;P=\text{0}.\text{4}\text{9}\text{7}$
The number of daily watching beauty contents	Z^*^ = 4.67, P < 0.001	$X^{\text{2}}=\text{1}\text{9}.\text{3}\text{7},\;P<\text{0}.\text{0}\text{0}\text{1}$

*: Standardized Mann-Whitney test, **: Tendency to cosmetic surgery score more than 72.

Linear regression, including demographic variables (students’ age as continuous, gender, marital status, and income as nominal, field of study and educational level as ordinal features), daily watching beauty-related content, perceived body image, and self‑esteem, was fitted as factors of the tendency toward cosmetic surgery. The interaction term of gender with body image (Wald X^2^ = 0.14, P-value = 0.711) and self-esteem (Wald X^2^ = 2.46, P-value = 0.0.117), is attached in supplementary files (S1 Table). Interaction terms were not significant. Finally, the linear regression without interaction terms was fitted and was statistically significant (F_9,186_ = 8.39, P < 0.001) and had good goodness-of-fit (Adjusted R-square = 0.25, Effect Size f^2^ = $\frac{R^{\text{2}}}{{\text{1}-\;R}^{\text{2}}}=\;$0.33) (Table 3).

**Table 3:** Output of the regression relationship between the tendency to undergo cosmetic surgery and demographic characteristics, body image, and self-esteem for all students.

Feature (Ref)	β (SE)	95% CI	t(p)	VIF
Age	−0.32(0.24)	−0.80,0.16	1.73(0.188)	1.51
Gender (male)	2.24(2.73)	−3.10,7.58	0.68(0.411)	1.06
Marital Status (married)	−1.89(4.54)	−10.78,7.00	0.17(0.677)	1.25
Field of study (S4)	–	–	4.69(0.196)	1.23
S1	5.16(3.25)	−1.22,11.53	2.52(0.113)	–
S2	7.29(3.38)	0.67,13.92	4.66(0.031)	–
S3	5.28(3.54)	−1.65,12.21	2.23(0.135)	–
Educational Level (MSc &PhD)	0.58(2.60)	−4.52,5.68	0.05(0.823)	1.31
Income (>300)	2.21(1.85)	−1.42,5.85	1.42(0.233)	1.06
Body image	−0.05(0.05)	−0.15,0.04	1.30(0.254)	1.02
Self-esteem	−1.25(0.23)	−1.70,-0.80	29.04(<0.001)	1.06
Watching beauty-related content (Rarely)	10.06(1.93)	13.83,6.28	27.29(<0.001)	1.06

Ref: Reference subgroups, β: Regression coefficient, SE: Standard deviation, VIF: Variance Inflation Factor, S1: Health & Basic Sciences, S2: Nursing & Midwifery, S3: Medicine, Pharmacy and Dentistry, S4: Emergency Medicine, Operating Room, and Anesthesia, Income: less than 300 million IRR and more than 300 million IRR.

Normal distribution, homogeneity of variance, and random variation of regression residuals are plotted in the supplementary file (S1–S4 Figs). The plots indicated that all of the mentioned assumptions were validated. The frequency of watching beauty content (direct relationship) and self‑esteem (inverse relationship) were statistically significant factors. Standardised coefficients of significant variables (frequency of watching beauty content = 0.31; self-esteem = 0.40) indicated that self-esteem and then viewing frequency were the most important factors in that order.

To assess the inverse role of self-esteem on the tendency to undergo cosmetic surgery, linear regression was fitted for gender separately (male: F_8,18_ = 2.43, Adjusted R^2^ = 0.31, femael:F_8,160_ = 8.98, Adjusted R^2^ = 0.28). Since 14% of participants were male, the interpretation of the coefficient in the regression with male participants should be interpreted with caution. The regression coefficients (P-values) for the frequency of watching beauty content (per day) were 10.87(<0.001) for women and 0.13(0.985) for men (Table 4), for a total of 10.06 (<0.001) (Table 3). Given that the relationship between the tendency to undergo cosmetic surgery and the self-esteem of female students was significant and non-significant for men, Fig 4 is drawn to show the distribution of the scores of the tendency to undergo cosmetic surgery and the self-esteem of students by gender.

**Table 4:** Output of the regression relationship between the tendency to undergo cosmetic surgery and demographic characteristics, body image, and self-esteem across students’ gender.

	Regression for females			Regression for males		
Feature (Ref)	β (SE)	95% CI	t(p)	β (SE)	95% CI	t(p)
Age	−0.46(0.25)	−0.95,0.04	3.28(0.070)	−0.08(0.88)	−1.79,1.64	0.00(0.926)
Marital Status (married)	−1.56(4.92)	−11.19,8.07	0.10(0.751)	4.03(15.44)	−26.22,34.29	0.07(0.794)
Field of study (s4)	–	–	4.03(0.258)	–	–	0.93(0.818)
S1	3.15(3.37)	−3.47,9.76	0.87(0.351)	7.33(11.01)	−14.26,28.91	0.44(0.506)
S2	6.48(3.48)	−0.34,13.30	3.46(0.063)	−0.61(10.09)	−20.38,19.17	0.00(0.952)
S3	4.22(3.75)	−3.13,11.58	1.27(0.260)	0.42(9.13)	17.47,18.32	0.00(0.963)
Educational Level (MSc &PhD)	0.87(2.76)	−4.55,6.29	0.10(0.752)	−10.00(6.85)	−23.43,3.42	2.13(0.144)
Income (>300)	1.62(1.96)	−2.22,5.45	0.68(0.409)	4.96(5.13)	−5.09,15.00	0.94(0.333)
Self-esteem	−1.23(0.24)	−1.69,-0.76	26.84(<0.001)	−1.53(0.81)	−3.12,0.06	3.58(0.059)
Body image	0.01(0.05)	−0.09,0.12	0.06(0.808)	−0.23(0.10)	−0.42,-0.04	5.44(0.020)
Watching beauty-related content (Rarely)	10.87(1.97)	14.74,7.00	30.35(<0.001)	0.13(7.20)	13.98,14.24	0.00(0.985)

Ref: Reference subgroups, β: Regression coefficient, SE: Standard deviation, S1: Health & Basic Sciences, S2: Nursing & Midwifery, S3: Medicine, Pharmacy and Dentistry, S4: Emergency Medicine, Operating Room, and Anesthesia, Income: less than 300 million IRR and more than 300 million IRR.

**Fig 4:**
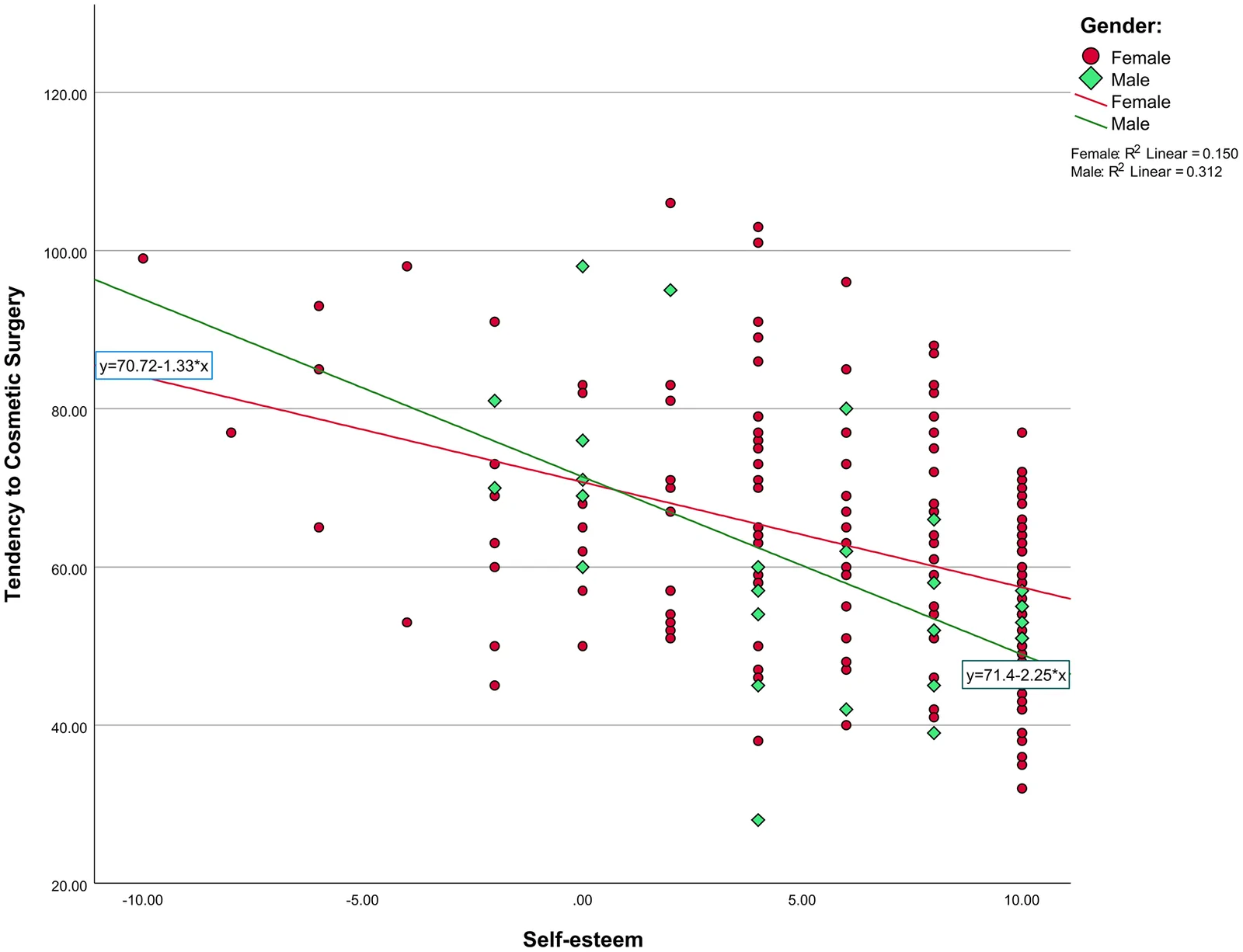
Dependency between the tendency to undergo cosmetic surgery and self-esteem, given the gender of the participants.

In Fig 4, it can be seen that the tendency to undergo cosmetic surgery among females, compared to male students, was significantly associated with their self-esteem.

## Discussion

This cross-sectional research among medical students found that several interrelated variables were significantly associated with the tendency to undergo cosmetic surgery, controlling for demographic variables. These variables included the extent of watching beauty-related media per day and self-esteem, which showed significant associations with the tendency to undergo cosmetic surgery. Among these variables, self-esteem showed the high level of association (standardized regression coefficient = 0.40), followed by daily media watching (standardized regression coefficient = 0.31). Students’ self-esteem was associated with the tendency to undergo cosmetic surgery in an inverse direction. The current research contributes to our understanding of the complex interplay between psychological and sociocultural factors that affect aesthetic preferences among young adults. The combination of these factors provides new insights into the complex process of decision-making about one’s appearance in a culture where access to and demand for cosmetic surgery were common.

### Gender ratio

This study found that 86% of participants were female, which aligns closely with the gender ratio at Iranian medical universities. Niroomand et al. reported nearly 80%, while Nasrabadi et al. reported 65%. Beyraghi and Soklaridis [25] reported that in 2003, 73% of students were female and tried to find psychological factors for this gender ratio. In conclusion, females were more than two-thirds of students at Iranian medical universities.

### Media exposure and interest in cosmetic surgery

Watching beauty content (with categories: rarely and usually) on social media is an important factor in inclination to cosmetic surgery, with a positive association. Holding other associated factors constant, students who watch beauty-related content on social media usually, were 10.06 times more likely to consider cosmetic surgery compared to those who rarely watch such content. This finding is consistent with the growing literature suggesting media exposure to content focused on appearance is associated with body dissatisfaction and intentions for altering one’s appearance. Social media, such as Instagram, TikTok, and Snapchat, continue to promote ideals of beauty through visual media. The continued exposure to such media may promote body concerns due to how one looks in public situations, thereby increasing interest in altering one’s physical appearance. Systematic reviews of literature have also found that exposure to ideals of visual aesthetics through social media is associated with negative body image and increased interest in cosmetic surgery across different categories of people [12].

Mechanisms for media exposure and interest in cosmetic surgery may also be attributed to appearance comparison. Individuals exposed to media promoting ideals of beauty may be more likely to compare their physical appearance to ideals, thereby increasing dissatisfaction and interest in cosmetic surgery. Studies conducted on adolescents and young adults found that exposure to selfies and comparisons of images mediated the association between media exposure and interest in cosmetic surgery, supporting the theory of social comparison as a cognitive mechanism for interest in cosmetic surgery [26].

### Body image dissatisfaction

The effect of body image on tendency to undergo cosmetic surgery was not significant in the present study. The orientation toward surgical enhancement in the presence of body dissatisfaction has been widely documented: objectification and sociocultural beauty norms contribute to internalized ideals that devalue individual bodies, increasing interest in aesthetic procedures [27]. Moreover, structural models integrating body perception suggest that body dissatisfaction can directly and indirectly influence surgical acceptance, with psychosocial variables such as relationship satisfaction and self-esteem serving as intermediaries [12].

In our context, this may indicate the influence of multi-level sociocultural pressures, especially where aesthetic norms are highly prominent. When individuals adopt high beauty standards, the gap between their actual and ideal appearance widens, with increasing emphasis on cosmetic changes being associated.

### Self-esteem

The most striking aspect of this study is the finding that people who have lower levels of self-esteem are more inclined to be interested in undergoing cosmetic surgery (negative regression coefficient). In other words, based on regression findings and after adjustment for other variables, a one-unit higher self-esteem score was associated with a 1.39-unit lower tendency to undergo cosmetic surgery in students.

This result is consistent with the traditional view that people who have lower levels of self-esteem may be more inclined to attempt to change their appearance (negative regression coefficient)

### Practical and educational implications

The findings of this study have several implications. One of these is the addition of media literacy and critical thinking courses to college curricula. This could enable young people to contextualize their perception of beauty, thus minimizing the risk of accepting negative beauty standards. There is sufficient evidence to show that media literacy courses reduce positive attitudes towards cosmetic procedures, improve body satisfaction, and boost self-esteem [28].

Another implication is for mental health practitioners to ask patients about their media consumption habits and how they measure up to other people when evaluating their reasons for undergoing cosmetic procedures. Differentiating between external social influences and intrinsic psychological needs is essential for effective counseling. Considering the pervasiveness of beauty-related content, it is suggested that health interventions focus on promoting body positivity and diverse body types to reduce the impact of the dominant standard of beauty.

### Strengths and limitations

The strength of this study is that it simultaneously measures exposure to the media, body image, and self-esteem as part of a comprehensive multivariable model. This will allow researchers to understand how these factors interact and work together to provide a better understanding of the subject. On the other hand, the study has certain limitations that need to be considered. First, this study is based on cross-sectional data; it is impossible to understand causality between any of these factors. The results can only indicate that the variables are associated. If researchers want to improve on these results, further studies will need to be longitudinal in nature. This will allow researchers to understand the interrelationship between exposure to the media, body image, and self-esteem over time. In detail, it would be important to understand whether or not the level of engagement with exposure to the media—passive viewing or active engagement—might act as a moderating factor on these relationships. Second, the tendency to undergo cosmetic surgery is a hidden feature, and participants’ responses to the questions may not be real, although the questions were designed to receive answers indirectly. As a result, this manner can cause an insignificant relationship between a feature as a dependent variable and demographic factors. Third, the authors of this study are informed that only 25% of the variation in tendency to cosmetic surgery is explained by the factors, and there are other features that can affect and explain the dependent variable. Maybe the insignificant relationship between the tendency to undergo cosmetic surgery in males and self-esteem is due to this topic and the small number of subgroups. Fourth, this study is a questionnaire-based one over an online platform (Porsline). Harman’s one- factor test revealed no serious problem with common method variance; the study depended only on the use of self-report surveys at one point in time. However, this suggests that some degree of common method bias might still be present. Fifth, findings of this study may not apply to students at non-medical universities and non-university youth. Given the growing use of social networks, their impact on youth behaviors may evolve over time, and this is another limitation of similar studies.

## Conclusion

The present study investigated the factors affecting the tendency towards cosmetic surgery among medical students in Khoy. The findings showed that frequent viewing of beauty-related content on media and social networks, as one of the important factors, was directly and significantly associated with an increased desire for cosmetic surgery. A noteworthy point in this study was the significant relationship between low self-esteem and a greater tendency to undergo cosmetic surgery, which is consistent with the third hypothesis of the study and the findings of the previous research. Overall, it can be concluded that psychological factors (such as self-esteem) and especially environmental media factors (such as watching beauty content) play a decisive role in shaping medical students’ attitudes towards cosmetic surgery. These findings emphasize the need to design and implement educational programs and preventive interventions in academic environments, with the aim of promoting media literacy, strengthening self-esteem, and providing a realistic understanding of the consequences of cosmetic surgery.

## Supporting information

S1 Table The results of linear regression with the interaction term of gender, body image, and self-esteem. (DOCX)

S1 Fig Histogram plot with a normal line to check the normal distribution of regression residuals. The histogram is bell-shaped and centered below the normal line.
(TIFF)

S2 Fig Normal Probability Plot to assess the normal distribution of residuals. The Normal P-P plot (S2 Fig) assesses how well the regression model residuals match a normal distribution. Since the points fall along the diagonal line, the PP plot and histogram indicate that the residuals are approximately normally distributed, so the model meets that key assumption.
(TIFF)

S3 Fig Regression standardized residuals VS. predicted number of the dependent variable for assessing homogeneity of variance. Since the range of dispersion of the standardized regression residuals is the same for different values of the predicted value of the dependent variable, the assumption of homogeneity of variance of the residuals is confirmed.
(TIFF)

S4 Fig Standardized regression residuals VS ID of participants to check about random variation of residuals. No clear pattern is observed in the participants’ regression residuals, suggesting that their magnitudes have random variations.
(TIFF)
