# Supplementary material for: A cross-sectional study of cosmetic surgery tendency and its psychological and media-related factors among medical students in 2024–2025
Source: PLoS One. 2026 Oct 30;21(10):e0360409. doi: 10.1371/journal.pone.0360409 (PMC13722214; doi:10.1371/journal.pone.0360409)
Supplement: Supplementary file 2 [file pone.0360409.s008.pdf]

**Dear Editor-in-Chief,**

**Dear Reviewers,**

Thank you for reviewing the corrections that were made to the manuscript previously, and thank you for the new comments. The authors of the manuscript will try to respond to the comments as much as possible.

Reviewer #2 :

1. Clarification of Rosenberg Self-Esteem Scale scoring and interpretation The finding that higher self-esteem is positively associated with cosmetic surgery tendency remains an important and unexpected result. Therefore, the manuscript should provide clearer information regarding the scoring procedure of the Rosenberg Self-Esteem Scale. It is advised to clarify whether reverse-scored items were appropriately recoded before calculating the total score and explicitly state whether higher scores represent higher self-esteem. This clarification is important because incorrect scoring could substantially affect the interpretation of the observed association.

> Response: Thanks for this comment. Again, we studied the questionnaire guide and noticed that self-esteem scores were not computed correctly. Lines 153-157 explain the rule of scoring. Then, we are very sorry for the mentioned computation error, and the entire manuscript has been corrected regarding the self-esteem results.

2. Clarification of beauty-related content exposure variable coding. The manuscript reports that frequency of watching beauty-related content is significantly associated with cosmetic surgery tendency; however, the coding approach used in the regression analysis remains unclear. It is need to specify whether this variable was treated as an ordinal/continuous variable or entered using dummy coding. If ordinal coding was applied, the authors should provide justification for assuming a linear relationship between exposure categories.

> Response: Thanks for the comment. Daily watching of beauty-related content is used as a categorical variable in three levels (never, 1-10 per day, and more than 10). The effect of these levels on the tendency to cosmetic surgery is explained in lines 235-239 and also in the regression in lines 295-300.

3. Consideration of unequal subgroup sizes for beauty-content exposureThe categories of beauty-related content exposure are highly unbalanced, with very small numbers of participants in some categories. This may influence the stability and interpretation of the statistical analyses. The authors should clarify whether any approach was used to address this issue (e.g., combining categories or sensitivity analysis). At minimum, this limitation should be discussed more explicitly.

> Response: Thanks for the comment. Daily watching beauty content was categorized into three levels. It's true that there are only 10 participants who have watched beauty-related content more than 10 times. But there is a note. Since combining the last two categories (1-10 and more than 10) can affect the severity of the feature, the authors were not advised. In other words, a student who watches beauty-related content 20 times will be in the same category as a student who watches it only 1 time !!! .

4. Avoidance of causal interpretation Although some improvements have been made, the manuscript still contains several statements that imply causality, which is not appropriate for a cross-sectional study design. For example, terms such as "increases," "strongest predictor," "drives," and "play a decisive role" should be replaced with more appropriate terms indicating association or relationship. The conclusions and discussion should be revised accordingly.

Response: Thanks. Corrected.

5. Interpretation of regression model findings The adjusted $R^2$ value of 0.21 indicates that the model explains approximately 21% of the variability in cosmetic surgery tendency. This level of explanatory power may be acceptable for psychosocial research; however, the manuscript should acknowledge more clearly that other unmeasured psychological, social, and environmental factors may contribute substantially to cosmetic surgery attitudes. Therefore, statements emphasizing the identified factors as decisive or comprehensive determinants should be moderated.

Response: Thanks so much. Lines 379-83 explain this topic.

6. Statistical reporting and transparency: Although the regression analysis has been improved, the authors should ensure complete reporting of regression parameters, including regression coefficients, confidence intervals, p-values, and standard errors. Clear and consistent reporting will improve reproducibility and interpretation of the findings.

Response: Thanks for the comment. Table 3 includes regression coefficients (95% confidence intervals), t-values, and p-values. When the regression coefficients and t-values are known, standard errors can be calculated using the formula $t = \beta / SE$. We attempted to include SE in Table 3 to address your comment, but space constraints prevented this.

7. Final language revision The manuscript has improved; however, several grammatical issues remain that affect readability and scientific clarity. A final professional English language editing is recommended before publication. For example, some sentences remain unclear.

Response: Thanks. Corrected. All of the text was reviewed again for typos and grammatical errors.

**Minor points:**

1. Terminology regarding predictors: The manuscript frequently uses the term "predictor" throughout the Results, Discussion, and Conclusion sections. Whileacceptable in statistical modeling, this terminology may imply prediction or causation. Please consider replacing it with "associated factor" or "independent correlate" where appropriate.

Response: Corrected.

2. Interpretation of self-esteem finding The discussion of the positive relationship between self-esteem and cosmetic surgery tendency has been improved; however, some statements remain unclear. The authors should avoid phrases such as "reverse direction" and instead directly explain that higher self esteem scores were statistically associated with higher cosmetic surgery tendency in this sample.

Response: Thanks for the comment. After score correction for self-esteem, there is an inverse relationship between self-esteem and the tendency toward cosmetic surgery, and the interpretation has been rephrased.

The authors of the manuscript, once again, say thanks for reviewing this manuscript and providing scientific feedback.

Good Luck.
