# Supplementary material for: A cross-sectional study of cosmetic surgery tendency and its psychological and media-related factors among medical students in 2024–2025
Source: PLoS One. 2026 Oct 30;21(10):e0360409. doi: 10.1371/journal.pone.0360409 (PMC13722214; doi:10.1371/journal.pone.0360409)
Supplement: Supplementary file 3 [file pone.0360409.s001.docx]

**Supplementary Materials:**

S1 table. The results of linear regression with the interaction term of gender, body image, and self-esteem.

| Features | F | P-value |
| --- | --- | --- |
| Age | 3.88 | 0.049 |
| Gender | 0.35 | 0.555 |
| Marital Status | 0.00 | 0.983 |
| Education field | 6.53 | 0.088 |
| Educational Level | 2.09 | 0.352 |
| Income | 0.96 | 0.326 |
| watching beauty content | 34.27 | <0.001 |
| Body image | 0.15 | 0.696 |
| Self-esteem | 22.24 | <0.001 |
| Gender ×Body-image | 0.14 | 0.711 |
| Gender × Self-esteem | 2.46 | 0.117 |

Since the P-value of the interaction terms was greater than 0.05, they were not significant.
