# Supplementary material for: A cross-sectional study of cosmetic surgery tendency and its psychological and media-related factors among medical students in 2024–2025
Source: PLoS One. 2026 Oct 30;21(10):e0360409. doi: 10.1371/journal.pone.0360409 (PMC13722214; doi:10.1371/journal.pone.0360409)
Supplement: Supplementary file 5 [file pone.0360409.s006.docx]

| **Date:** | Jan 20 2026 06:39AM |
| --- | --- |
| **To:** | "Hassan Ebrahimpour Sadagheyani" sadageyani@yahoo.com |
| **From:** | "PLOS ONE" plosone@plos.org |
| **Subject:** | PLOS ONE Decision: PONE-D-25-56736 |

PONE-D-25-56736
An Investigation into the Tendency toward Cosmetic Surgery among Medical Students and Its Relationship with Demographic Characteristics, Watching Beauty-Related Content, Body Image, and Self-Esteem
PLOS One

Dear Dr. Ebrahimpour Sadagheyani,

Thank you for submitting your manuscript to PLOS ONE. After careful consideration, we have decided that your manuscript does not meet our criteria for publication and must therefore be rejected.

The manuscript has been assessed by two reviewers, and their comments are appended below.

The reviewers have raised major concerns about the study regarding its scientific depth and methodological rigour.

In light of the concerns identified, the manuscript does not meet PLOS ONE’s criteria for publication in its current form.

I appreciate that you will be disappointed by this decision and I am sorry that we cannot be more positive on this occasion, but hope that you appreciate the reasons for this decision.

Kind regards,

Ilse Bloom
Staff Editor
PLOS One

Additional Editor Comments (if provided):

[Note: HTML markup is below. Please do not edit.]

Reviewers' comments:

Reviewer's Responses to Questions

**Comments to the Author**

1. Is the manuscript technically sound, and do the data support the conclusions?

The manuscript must describe a technically sound piece of scientific research with data that supports the conclusions. Experiments must have been conducted rigorously, with appropriate controls, replication, and sample sizes. The conclusions must be drawn appropriately based on the data presented.

Reviewer #1: Partly

Reviewer #2: Partly

2. Has the statistical analysis been performed appropriately and rigorously?

 Reviewer #1: No

Reviewer #2: Yes

3. Have the authors made all data underlying the findings in their manuscript fully available?

The [PLOS Data policy](http://www.plosone.org/static/policies.action#sharing) requires authors to make all data underlying the findings described in their manuscript fully available without restriction, with rare exception (please refer to the Data Availability Statement in the manuscript PDF file). The data should be provided as part of the manuscript or its supporting information, or deposited to a public repository. For example, in addition to summary statistics, the data points behind means, medians and variance measures should be available. If there are restrictions on publicly sharing data—e.g. participant privacy or use of data from a third party—those must be specified.

 Reviewer #1: Yes

Reviewer #2: Yes

 4. Is the manuscript presented in an intelligible fashion and written in standard English?

PLOS ONE does not copyedit accepted manuscripts, so the language in submitted articles must be clear, correct, and unambiguous. Any typographical or grammatical errors should be corrected at revision, so please note any specific errors here.

 Reviewer #1: Yes

Reviewer #2: No

 5. Review Comments to the Author

Please use the space provided to explain your answers to the questions above. You may also include additional comments for the author, including concerns about dual publication, research ethics, or publication ethics. (Please upload your review as an attachment if it exceeds 20,000 characters)

Reviewer #1: After careful evaluation of the full manuscript, I have significant concerns regarding the study’s scientific contribution and suitability for publication.
While the associations explored are relevant, the study in its current form lacks rigor and impact.

1. The variables (body image, self-esteem, media exposure vs. cosmetic surgery interest) are well established in the existing literature across multiple populations.

Author's Response: We would like to express our thanks for the reviewer’s interest in the novelty of the selected variables. The variables of body image, self-esteem, and media exposure have been studied individually before. However, we have revised the manuscript to highlight the contribution of the current study (Introduction, paragraph before study hypothesis). The current study extends the previous studies on body image, self-esteem, and media exposure, as we have studied the variables simultaneously for a medically educated population, who are an understudied group with high health literacy and sociocultural body image pressures. We have made appropriate revisions to the Introduction section of the manuscript.

2. The study does not advance understanding, mechanisms, or implications. It is unclear how the results may impact medical education, mental health interventions, media literacy, or directions for future research.
Author's Response: We would like to express our gratitude for the reviewer’s emphasis on the importance of the theoretical foundation and implications. We have made significant changes to the Discussion section. First, we have contextualized the findings from the perspective of Social Comparison Theory and self-enhancement processes that may underlie the relationship between media exposure and self-esteem. Second, we have clarified how the counterintuitive positive relationship between self-esteem and cosmetic surgery tendency may actually tap into optimization processes, as opposed to compensatory processes. Lastly, we have made significant modifications to the implications section, which include specific suggestions for the development of the medical education curriculum, mental health screening of students, and media literacy programs.

Advice to the Authors:
1. Increase sample size and provide a clear justification for the chosen sample, including power considerations and representativeness

Author's response: We would like to express our thanks to the reviewer for pointing out the importance of justifying the adequacy of samples. We have made significant modifications to the manuscript, stating the statistical parameters used for determining the sample size (alpha = 0.05, power = 0.80, effect size f^2^ = 0.27). The response rate of 89% is adequate, thereby minimizing non-response bias. The effect size is of medium-to-large magnitude, indicating that the study is likely powered appropriately.

2. Strengthen methodological rigor: study design, handling of confounders, justification of statistical approach

Author Response: We improved the methodological part by specifically stating the assumptions of regression (normality of residuals, homoscedasticity, VIF values, and Durbin-Watson statistics). In addition, we investigated gender interaction effects to see if correlations were different across subgroups.

3. Discussion should not restate prior findings, but rather advance them

Author's responses: The Discussion has been revised totally to avoid repetition of results.

4. Provide stronger theoretical grounding and explain how it advances current knowledge.

Author's responses: added and corrected.

Reviewer #2: The topic is appropriate, the dataset is usable, and the analyses are largely sound. However, due to methodological inconsistencies, data reporting errors, and conceptual weaknesses, the article cannot be accepted unless these aspects are addressed.

General Assessment
This manuscript addresses a relevant and timely topic: the psychological and media-related determinants of cosmetic surgery inclination among medical students. Given the global rise in cosmetic procedures and the influence of social media, the research question is appropriate and fits within the scope of PLOS ONE.
The study benefits from:
• Use of validated instruments
• Ethical approval and informed consent
• A clearly stated set of hypotheses
• Appropriate statistical techniques for a cross-sectional design

Major Concerns (Must Be Addressed)
1. Sampling Method and Generalizability
• The study claims “random selection” in the abstract, but the Methods clearly state convenience (non-probability) sampling via social media.
• This is a serious inconsistency and must be corrected.
• The limitations of convenience sampling and its impact on external validity should be explicitly discussed.
Required action:
Clarify sampling throughout the manuscript and add a stronger limitations section.

Author's Response: We would like to thank the reviewer for pointing out this discrepancy. The phrase "random selection" was removed from the Abstract and corrected to reflect that it was a convenience (non-probability) sampling method using an online platform. Moreover, the Limitations section has been improved to include an acknowledgment of the possibility of selection bias and reduced external validity.

2. Gender Distribution Error
• There is a major inconsistency in reporting gender:
o Abstract: 86% female
o Table 1: 86% male (169 males, 27 females)
This directly affects:
• Interpretation of gender-stratified regression
• Conclusions regarding self-esteem differences
Required action:
Correct and verify gender data across the entire manuscript. This is critical.
Author's Response: We sincerely thank the reviewer for detecting this reporting inconsistency. After re-verifying the dataset, we corrected the gender distribution across the Abstract, Tables, and Results sections. The corrected distribution is 86% female and 14% male. All gender-stratified analyses were rechecked, and results remain statistically consistent. We apologize for this reporting error.

3. Conceptual Interpretation of Self-Esteem Findings
• The finding that higher self-esteem predicts higher cosmetic surgery tendency contradicts much of the literature and the authors’ own hypothesis.
• While the Discussion attempts to explain this as “self-enhancement,” the argument remains speculative and underdeveloped.
Required action:
• Strengthen theoretical justification using established frameworks (e.g., self-enhancement theory, consumer identity theory).
• Acknowledge alternative explanations (e.g., confidence to pursue elective procedures, socioeconomic confounding).

Author's Response: We both agree that a more solid theoretical foundation was necessary for the interpretation of the self-esteem finding. By adding viewpoints on consumer identity and self-enhancement theory, we have broadened the discussion. Alternative explanations, such as socioeconomic factors and motivations for elective health behavior, are also acknowledged.

4. Measurement and Interpretation Issues
• Rosenberg Self-Esteem Scale scoring is unconventional here (−10 to +10), yet results are treated as linear without sufficient justification.

Author's Response: In line with earlier regional validation studies, the Rosenberg scale was scored using a bipolar scoring system (-1 to +1). After confirming the normalcy assumptions, the continuous total score was kept for regression analysis.

• The interpretation of regression coefficients (e.g., “for every ten contents watched”) is unclear and potentially misleading.
Required action:
• Clarify scaling, units, and assumptions.
• Avoid causal or quasi-causal language in a cross-sectional study.

Authors Response: This interpretation was in the discussion section. Since all of the discussion revised and rewritten, this mistake has been corrected.

5. Data Availability Statement (Non-Compliance with PLOS ONE Policy)
• “Data available via official request or email” does not meet PLOS ONE requirements.
• No justification for restriction is provided.
Required action:
• Deposit anonymized data in a public repository or provide a valid ethical/legal reason for restriction.

Author's Response: A section about the availability of data was added to the paper.

Minor Concerns (Must Be Addressed)
• English language editing is needed (grammar, article use, sentence flow).

Done
• Some references are outdated or inconsistently formatted.

Author's Response: Some new references have been added, and some of them have been changed. Finally, Outdated references are related to questionnaire or reliability studies.

• Redundant explanation of normality plots (histograms not essential for publication).

• Figures add limited value and could be reduced.

Author's Response: Histograms alongside the normality check indicate the dispersion of the main factors influencing the tendency toward cosmetic surgery, particularly the variation of these factors by gender.

• Some claims in the Discussion are overly strong given the study design.

Author's Response: Discussen rewrited. Please review again.

Ethical Considerations
No major ethical violations identified. However:
• Clarify how anonymity was preserved given online data collection.
• Confirm that consent included publication of anonymized data.

Author's Response: Corrected.

6. PLOS authors have the option to publish the peer review history of their article ([what does this mean?](https://journals.plos.org/plosone/s/editorial-and-peer-review-process#loc-peer-review-history)). If published, this will include your full peer review and any attached files.


If you choose “no”, your identity will remain anonymous but your review may still be made public.


**Do you want your identity to be public for this peer review?** For information about this choice, including consent withdrawal, please see our [Privacy Policy](https://www.plos.org/privacy-policy).

Reviewer #1: No

Reviewer #2: No

[NOTE: If reviewer comments were submitted as an attachment file, they will be attached to this email and accessible via the submission site. Please log into your account, locate the manuscript record, and check for the action link "View Attachments". If this link does not appear, there are no attachment files.]

- - - - -
For journal use only: PONEDEC3
