# Supplementary material for: A cross-sectional study of cosmetic surgery tendency and its psychological and media-related factors among medical students in 2024–2025
Source: PLoS One. 2026 Oct 30;21(10):e0360409. doi: 10.1371/journal.pone.0360409 (PMC13722214; doi:10.1371/journal.pone.0360409)
Supplement: Supplementary file 6 [file pone.0360409.s009.docx]

Dear Editor-in-Chief; Dear Reviewer

Thank you for reviewing this manuscript in deep. The last uploaded manuscript, compared to the initial manuscript, has more than 90% of its content corrected. Perhaps, like me, you believe that the published manuscript contained some errors. The authors of this manuscript tried to correct errors as well as possible. But some errors can not be corrected. Response to comments:

1- Coding and interpretation of the beauty-related content exposure variable:

Thanks. Tendency toward cosmetic surgery is scored based on questionnaire items (24-120). The cut-off of 72 is the median of the scale scores (24 + 120/2 = 72). Participants with scores more than the median are susceptible to cosmetic surgery. This point was added to the scale explanation in the manuscript (Lines 158-62).

2- The potential effect of unequal subgroup sizes:

Thanks. The unequal sample sizes by gender of students are explained in lines 284-89. This section reviewed similar studies with the same gender ratio.

**Dear reviewer**: All of the authors of this manuscript are teachers at Khoy University of Medical Sciences and are informed about the gender ratio of males to females in other medical universities. This gender ratio is real in the studied population, especially among medical students. It's a fact that the male-to-female student ratio in some medical courses in Iran (especially Nursing, Public Health, Environmental Health, Laboratory sciences, …) is less than 10%.

3- The continued use of causal or overly strong language in a cross-sectional study, and the completeness of statistical reporting:

Thanks. The authors have tried to prevent causal interpretation of results. Another time, interpretations are checked.

4- Following the correction of the Rosenberg Self-Esteem Scale scoring, the authors should also ensure that the revised direction and interpretation of the self-esteem findings are fully consistent throughout the Abstract, Results, Discussion, Conclusion, tables, and supplementary material.

Thanks. Done.

5- Some grammatical and stylistic issues also remain and require final English language editing.

Thanks. Grammatical errors will be checked again.

Good Luck.
