# Supplementary material for: A cross-sectional study of cosmetic surgery tendency and its psychological and media-related factors among medical students in 2024–2025
Source: PLoS One. 2026 Oct 30;21(10):e0360409. doi: 10.1371/journal.pone.0360409 (PMC13722214; doi:10.1371/journal.pone.0360409)
Supplement: Supplementary file 7 [file pone.0360409.s010.docx]

**Dear Editor-in-Chief; Dear Reviewer #2**

Thanks again for reviewing this manuscript.

Reviewer #2: The authors have addressed several concerns raised in the previous round, and the manuscript has improved. However, the following issues still require clarification or correction before publication:

1. Please clarify whether the three exposure categories were entered into the regression using dummy/indicator coding or ordinal coding. The reported coefficient of 7.78 should also be interpreted appropriately for multiple linear regression rather than as “7.78 times more likely.”

Response: Thanks for the comment. Lines 247-50 :

Linear regression, including demographic variables (students' age as continuous, gender, marital status, and income as nominal, field of study and educational level as ordinal features), daily watching beauty-related content, perceived body image, and self‑esteem was fitted as factors of the tendency toward cosmetic surgery.

2. The beauty-content exposure categories remain highly unbalanced, particularly the highest-exposure group, which contains only a small number of participants. Please discuss how this imbalance may affect the stability and reliability of the regression estimates and the interpretation of this association. If no sensitivity analysis was performed, this should at least be acknowledged as a limitation.

Response: Thanks. Beauty-content exposure is categorized at two levels (Table 1: rarely (n = 120) and usually (n= 76)).

3. The corrected analysis reports an unstandardized coefficient of −1.39 for self-esteem (95% CI: −1.86 to −0.92, P<0.001), indicating an inverse association with cosmetic surgery tendency, while the manuscript says: "self-esteem showed the strongest association (standardized regression coefficient = 0.40)" and then says media watching was 0.31. Please ensure that this revised result is consistently reflected in the Abstract, Results, Discussion, Conclusion, tables, and supplementary material, and that any values or interpretations from the previous analysis have been removed. The authors should also verify that statements regarding the relative strength of this association are based on the corrected standardized coefficients.

Response: Thanks. Done and checked.

4. Standard errors are still not reported in the regression table. Please include them either in the main table or supplementary material, and verify that all reported regression coefficients and interpretations correspond to the corrected analysis.

Response: Tables 3 and 4 redesigned, and SE is added.

5. Some causal or overly strong expressions remain, such as “strong factor,” “increasing interest,” and “strongest association.” These should be revised to reflect associations only, given the cross-sectional design. Several grammatical and stylistic problems remain in the revised manuscript, including duplicated wording. A final professional English-language edit is recommended before publication.
Response: Thanks again. Expression is corrected, and an English-language edit has been done.

Good Luck.
