# Supplementary material for: A cross-sectional study of cosmetic surgery tendency and its psychological and media-related factors among medical students in 2024–2025
Source: PLoS One. 2026 Oct 30;21(10):e0360409. doi: 10.1371/journal.pone.0360409 (PMC13722214; doi:10.1371/journal.pone.0360409)
Supplement: Peer review history

# Author comment: Author response to Decision Letter 0

**DOI:** 10.1371/journal.pone.0360409.r001

1 May 2026

Attachment Submitted filename: 04-Reponse to Reviewers.docx

# Peer review: Decision Letter 0

**DOI:** 10.1371/journal.pone.0360409.r002

**Authors:** Muhammad Athar

1 Jul 2026

Dear Dr. Ebrahimpour Sadagheyani,

Thank you for submitting your manuscript to PLOS ONE. After careful consideration, we feel that it has merit but does not fully meet PLOS ONE’s publication criteria as it currently stands. Therefore, we invite you to submit a revised version of the manuscript that addresses the points raised during the review process.

Please submit your revised manuscript by Aug 30 2026 11:59PM. If you will need more time than this to complete your revisions, please reply to this message or contact the journal office at plosone@plos.org. When you're ready to submit your revision, log on to https://www.editorialmanager.com/pone/ and select the 'Submissions Needing Revision' folder to locate your manuscript file.

- A letter that responds to each point raised by the academic editor and reviewer(s). You should upload this letter as a separate file labeled 'Response to Reviewers'.
- A marked-up copy of your manuscript that highlights changes made to the original version. You should upload this as a separate file labeled 'Revised Manuscript with Track Changes'.
- An unmarked version of your revised paper without tracked changes. You should upload this as a separate file labeled 'Manuscript'.

If you would like to make changes to your financial disclosure, please include your updated statement in your cover letter. Guidelines for resubmitting your figure files are available below the reviewer comments at the end of this letter.

If applicable, we recommend that you deposit your laboratory protocols in protocols.io to enhance the reproducibility of your results. Protocols.io assigns your protocol its own identifier (DOI) so that it can be cited independently in the future. For instructions see: https://journals.plos.org/plosone/s/submission-guidelines#loc-laboratory-protocols. Additionally, PLOS ONE offers an option for publishing peer-reviewed Lab Protocol articles, which describe protocols hosted on protocols.io. Read more information on sharing protocols at https://plos.org/protocols?utm_medium=editorial-email&utm_source=authorletters&utm_campaign=protocols.

As the corresponding author, your ORCID iD is verified in the submission system and will appear in the published article. PLOS supports the use of ORCID, and we encourage all coauthors to register for an ORCID iD and use it as well. Please encourage your coauthors to verify their ORCID iD within the submission system before final acceptance, as unverified ORCID iDs will not appear in the published article. *Only*  the individual author can complete the verification step; PLOS staff *cannot*  verify ORCID iDs on behalf of authors.

We look forward to receiving your revised manuscript.

Kind regards,

Muhammad Athar, PhD

Academic Editor

PLOS One

Journal Requirements:

When submitting your revision, we need you to address these additional requirements.

1.Please ensure that your manuscript meets PLOS ONE's style requirements, including those for file naming. The PLOS ONE style templates can be found at

https://journals.plos.org/plosone/s/file?id=wjVg/PLOSOne_formatting_sample_main_body.pdf and

https://journals.plos.org/plosone/s/file?id=ba62/PLOSOne_formatting_sample_title_authors_affiliations.pdf

2. Please provide additional details regarding participant consent. In the ethics statement in the Methods and online submission information, please ensure that you have specified (1) whether consent was informed and (2) what type you obtained (for instance, written or verbal, and if verbal, how it was documented and witnessed). If your study included minors, state whether you obtained consent from parents or guardians. If the need for consent was waived by the ethics committee, please include this information.

If you are reporting a retrospective study of medical records or archived samples, please ensure that you have discussed whether all data were fully anonymized before you accessed them and/or whether the IRB or ethics committee waived the requirement for informed consent. If patients provided informed written consent to have data from their medical records used in research, please include this information.

3. For studies involving third-party data, we encourage authors to share any data specific to their analyses that they can legally distribute. PLOS recognizes, however, that authors may be using third-party data they do not have the rights to share. When third-party data cannot be publicly shared, authors must provide all information necessary for interested researchers to apply to gain access to the data. (https://journals.plos.org/plosone/s/data-availability#loc-acceptable-data-access-restrictions)

For any third-party data that the authors cannot legally distribute, they should include the following information in their Data Availability Statement upon submission:

1) A description of the data set and the third-party source

2) If applicable, verification of permission to use the data set

3) Confirmation of whether the authors received any special privileges in accessing the data that other researchers would not have

4) All necessary contact information others would need to apply to gain access to the data

4. Your ethics statement should only appear in the Methods section of your manuscript. If your ethics statement is written in any section besides the Methods, please delete it from any other section.

5. Please upload a copy of Figure 4, to which you refer in your text on page 8. If the figure is no longer to be included as part of the submission please remove all reference to it within the text.

6. If the reviewer comments include a recommendation to cite specific previously published works, please review and evaluate these publications to determine whether they are relevant and should be cited. There is no requirement to cite these works unless the editor has indicated otherwise.

[Note: HTML markup is below. Please do not edit.]

Reviewers' comments:

Reviewer's Responses to Questions

**Comments to the Author**

1. Is the manuscript technically sound, and do the data support the conclusions?

Reviewer #1: Partly

Reviewer #2: Partly

**********

2. Has the statistical analysis been performed appropriately and rigorously?

Reviewer #1: Yes

Reviewer #2: No

**********

3. Have the authors made all data underlying the findings in their manuscript fully available?

The PLOS Data policy

Reviewer #1: Yes

Reviewer #2: Yes

**********

4. Is the manuscript presented in an intelligible fashion and written in standard English?

Reviewer #1: Yes

Reviewer #2: No

**********

Reviewer #1: The manuscript has publication potential, but substantial revisions are required before it can be considered for acceptance. If the authors adequately address the raised concerns and strengthen the theoretical contribution, the paper may become suitable for publication.

Major Comments:

1. The study employed convenience sampling through an online platform and social media recruitment. However, it is expected that participants with greater interest in beauty-related issues may have been more likely to respond. The authors should discuss how this may have influenced the observed associations and limit claims of generalizability accordingly.

2. The sample consists of considerably higher percentage of females than males, raising concerns regarding representativeness and the validity of the analyses. The authors should justified the reason about this issue.

3. The regression model in this manuscript explains only 21% of the variance. The authors should discuss that why many important determinants were not measured.

4. The manuscript treated "frequency of watching beauty content" as a continuous predictor despite being collected in ordinal categories. The authors should justify this analytical choice or use appropriate dummy coding.

5. All variables were collected using self-reported questionnaires at a single time point. The manuscript does not address the possibility of common method variance inflating observed correlations.

6. The manuscript reports Cronbach's alpha values from previous studies but does not provide reliability coefficients for the current dataset. Internal consistency statistics should be reported for all scales used in this sample.

7. Fifteen percent of participants had already undergone cosmetic procedures. Previous surgery experience could substantially influence attitudes toward future cosmetic surgery and should have been considered as a moderator or explored in greater depth.

Minor Comments:

1. The manuscript reports that 220 students viewed the questionnaire and 196 completed it, yet the Results section states that 250 students studied the questionnaire. Please report the correct information.

2. In Table 2, the ANOVA result for beauty-content exposure is reported as F = 2.63 with P < 0.001, which appears inconsistent and should be verified.

3. Although the authors argue that studying medical students is novel, the relationships among body image, self-esteem, media exposure, and cosmetic surgery intentions are already extensively documented. The novelty of the manuscript should be clearly explained.

Reviewer #2: Comments to the Authors

The study investigates an interesting and relevant topic regarding the psychological and social predictors of cosmetic surgery tendency among medical students. The dataset is adequate, and the use of validated instruments and regression analysis is appropriate. However, the manuscript requires major revision before it can be considered for publication. Several methodological, statistical, and reporting issues should be addressed to improve the scientific rigor, clarity, and overall quality of the manuscript.

1. Please strengthen the novelty statement in the Introduction by clearly explaining how this study advances the existing literature beyond previous studies investigating body image, self-esteem, and media exposure in relation to cosmetic surgery.

2. The sample was recruited using convenience sampling through social media, which may introduce selection bias. Please discuss its impact on the representativeness and generalizability of the findings more explicitly in the limitations section.

3. The sample is predominantly female (86%), while only 27 male participants were included. Please discuss how this gender imbalance may have influenced the findings, particularly the gender-specific regression analyses.

4. The rationale for the sample size calculation should be clarified, as it appears to be based on an odds ratio from a previous study, whereas the primary analysis in this study is multiple linear regression.

5. Please clarify the coding and interpretation of the Rosenberg Self-Esteem Scale, particularly the handling of reverse-scored items, as the observed positive association between self-esteem and cosmetic surgery tendency differs from many previous reports.

6. The finding that higher self-esteem is associated with greater cosmetic surgery tendency is interesting but counterintuitive compared to much of the literature. However, there is a possible inconsistency in interpretation, as: Regression coefficient for self-esteem is positive (β = 1.38), yet the discussion describes a “reverse direction” interpretation.

7. The manuscript occasionally uses causal language (e.g., "increases", "drives", "promotes"). Since this is a cross-sectional study, causal interpretations should be avoided and replaced with statements indicating associations.

8. The description of the regression model in the Statistical Analysis section should be revised. The predictor variables are incorrectly referred to as dependent variables.

9. Please provide additional details of the regression analysis, including 95% confidence intervals, standard errors, and adjusted R² values, to improve the transparency and completeness of the statistical reporting.

10. Please clarify how the beauty-content exposure variable was coded in the regression model (continuous, ordinal, or dummy variables) and justify the selected approach.

11. Several reported values require verification for consistency. For example, the participant numbers (220 vs. 250), cosmetic surgery history (29 vs. 30 participants), and some statistical values in Table 2 appear inconsistent.

12. The reported F-statistic and corresponding P-value for family income in Table 2 appear unusual and should be verified to ensure accurate statistical reporting.

13. The categories for daily beauty-content exposure are highly unbalanced (Never = 120, 1–10 = 66, 11–20 = 6, >20 = 4). The authors should discuss whether these small subgroup sizes could affect the robustness of the ANOVA and regression analyses or consider combining categories where appropriate.

14. Figure 4 is discussed in the Results section but is not included in the manuscript. Please include the missing figure or remove the corresponding text.

15. The regression analysis performed separately for male participants should be interpreted cautiously because of the small male sample size, which may result in unstable regression estimates.

16. The regression model explains approximately 21% of the variance in cosmetic surgery tendency. Please acknowledge that additional unmeasured factors may contribute to the outcome and avoid overstating the predictive ability of the model.

17. The manuscript currently contains two separate limitation sections. These should be merged into a single comprehensive section to improve organization and readability.

18. The manuscript would benefit from careful English language editing to improve grammar, sentence structure, and overall readability.

19. Please ensure that the manuscript complies with the journal's requirements regarding data availability and reporting.

Minor Comments

1. Lines 109–110: The sentence "Kasmaei et al. used." is incomplete and should be rewritten to clearly describe the sample size calculation procedure.

2. Lines 159–160: The manuscript states that 250 students studied the questionnaire, whereas the Methods section reports that 220 students viewed the questionnaire. Please correct this inconsistency.

3. Lines 176–180: The statement "As regards gender distribution, the data..." is unclear because the presented histograms evaluate variable distributions rather than gender distribution. Please revise this sentence.

4. Lines 214–215: The sentence "Plotts indicated that all of the mentioned were validated" contains typographical and grammatical errors. It should be rewritten in clear scientific English.

5. Lines 271–272: The sentence ending with "making cosmetic." appears incomplete or truncated. Please revise this sentence.

6. Lines 273–285: The discussion regarding the positive association between self-esteem and cosmetic surgery tendency would benefit from citing additional recent studies that reported similar findings to better support this unexpected result.

7. Lines 321–323: The manuscript states that the self-esteem finding contradicts the first hypothesis. However, according to the Introduction, self-esteem corresponds to Hypothesis 3, not Hypothesis 1. Please correct this inconsistency.

8. Lines 334–338: A separate "Limitation" section is presented after the Conclusion, although limitations have already been discussed earlier. Consider merging both sections to improve manuscript organization and avoid repetition.

**********

what does this mean?). If published, this will include your full peer review and any attached files.

If you choose “no”, your identity will remain anonymous but your review may still be made public.

**Do you want your identity to be public for this peer review?**  For information about this choice, including consent withdrawal, please see our Privacy Policy

Reviewer #1: No

Reviewer #2: No

**********

[NOTE: If reviewer comments were submitted as an attachment file, they will be attached to this email and accessible via the submission site. Please log into your account, locate the manuscript record, and check for the action link "View Attachments". If this link does not appear, there are no attachment files.]

To ensure your figures meet our technical requirements, please review our figure guidelines: https://journals.plos.org/plosone/s/figures

You may also use PLOS’s free figure tool, NAAS, to help you prepare publication quality figures: https://journals.plos.org/plosone/s/figures#loc-tools-for-figure-preparation.

NAAS will assess whether your figures meet our technical requirements by comparing each figure against our figure specifications.

# Author comment: Author response to Decision Letter 1

**DOI:** 10.1371/journal.pone.0360409.r003

25 Jul 2026

Dear reviewers of the manuscript

The authors sincerely thank the reviewers for their valuable comments on various sections of the manuscript. They apologize for any typos and appreciate the methodological feedback once again.

Good Luck.

Attachment Submitted filename: informed consent.pdf

# Peer review: Decision Letter 1

**DOI:** 10.1371/journal.pone.0360409.r004

**Authors:** Muhammad Athar

10 Aug 2026

Dear Dr. Ebrahimpour Sadagheyani,

Thank you for submitting your manuscript to PLOS ONE. After careful consideration, we feel that it has merit but does not fully meet PLOS ONE’s publication criteria as it currently stands. Therefore, we invite you to submit a revised version of the manuscript that addresses the points raised during the review process.

Please submit your revised manuscript by Sep 24 2026 11:59PM. If you will need more time than this to complete your revisions, please reply to this message or contact the journal office at plosone@plos.org. When you're ready to submit your revision, log on to https://www.editorialmanager.com/pone/ and select the 'Submissions Needing Revision' folder to locate your manuscript file.

- A letter that responds to each point raised by the academic editor and reviewer(s). You should upload this letter as a separate file labeled 'Response to Reviewers'.
- A marked-up copy of your manuscript that highlights changes made to the original version. You should upload this as a separate file labeled 'Revised Manuscript with Track Changes'.
- An unmarked version of your revised paper without tracked changes. You should upload this as a separate file labeled 'Manuscript'.

If you would like to make changes to your financial disclosure, please include your updated statement in your cover letter. Guidelines for resubmitting your figure files are available below the reviewer comments at the end of this letter.

If applicable, we recommend that you deposit your laboratory protocols in protocols.io to enhance the reproducibility of your results. Protocols.io assigns your protocol its own identifier (DOI) so that it can be cited independently in the future. For instructions see: https://journals.plos.org/plosone/s/submission-guidelines#loc-laboratory-protocols. Additionally, PLOS ONE offers an option for publishing peer-reviewed Lab Protocol articles, which describe protocols hosted on protocols.io. Read more information on sharing protocols at https://plos.org/protocols?utm_medium=editorial-email&utm_source=authorletters&utm_campaign=protocols.

As the corresponding author, your ORCID iD is verified in the submission system and will appear in the published article. PLOS supports the use of ORCID, and we encourage all coauthors to register for an ORCID iD and use it as well. Please encourage your coauthors to verify their ORCID iD within the submission system before final acceptance, as unverified ORCID iDs will not appear in the published article. *Only*  the individual author can complete the verification step; PLOS staff *cannot*  verify ORCID iDs on behalf of authors.

We look forward to receiving your revised manuscript.

Kind regards,

Muhammad Athar, PhD

Academic Editor

PLOS One

Journal Requirements:

If the reviewer comments include a recommendation to cite specific previously published works, please review and evaluate these publications to determine whether they are relevant and should be cited. There is no requirement to cite these works unless the editor has indicated otherwise.

Please review your reference list to ensure that it is complete and correct. If you have cited papers that have been retracted, please include the rationale for doing so in the manuscript text, or remove these references and replace them with relevant current references. Any changes to the reference list should be mentioned in the rebuttal letter that accompanies your revised manuscript. If you need to cite a retracted article, indicate the article’s retracted status in the References list and also include a citation and full reference for the retraction notice.

[Note: HTML markup is below. Please do not edit.]

Reviewers' comments:

Reviewer's Responses to Questions

**Comments to the Author**

Reviewer #1: All comments have been addressed

Reviewer #2: (No Response)

**********

2. Is the manuscript technically sound, and do the data support the conclusions?

Reviewer #1: Yes

Reviewer #2: Partly

**********

3. Has the statistical analysis been performed appropriately and rigorously?

Reviewer #1: Yes

Reviewer #2: Yes

**********

4. Have the authors made all data underlying the findings in their manuscript fully available?

The PLOS Data policy

Reviewer #1: (No Response)

Reviewer #2: Yes

**********

5. Is the manuscript presented in an intelligible fashion and written in standard English?

Reviewer #1: Yes

Reviewer #2: No

**********

Reviewer #1: (No Response)

Reviewer #2: Comments to the Authors (Second Round Review):

The authors have revised the manuscript and have addressed many of the concerns raised in the previous review. The manuscript has therefore improved considerably. However, some issues related to statistical clarification, interpretation of findings, and reporting quality still require clarification. Addressing these points would further strengthen the scientific rigor and clarity of the manuscript.

1. Clarification of Rosenberg Self-Esteem Scale scoring and interpretation

The finding that higher self-esteem is positively associated with cosmetic surgery tendency remains an important and unexpected result. Therefore, the manuscript should provide clearer information regarding the scoring procedure of the Rosenberg Self-Esteem Scale. It is advised to clarify whether reverse-scored items were appropriately recoded before calculating the total score and explicitly state whether higher scores represent higher self-esteem. This clarification is important because incorrect scoring could substantially affect the interpretation of the observed association.

2. Clarification of beauty-related content exposure variable coding

The manuscript reports that frequency of watching beauty-related content is significantly associated with cosmetic surgery tendency; however, the coding approach used in the regression analysis remains unclear. It is need to specify whether this variable was treated as an ordinal/continuous variable or entered using dummy coding. If ordinal coding was applied, the authors should provide justification for assuming a linear relationship between exposure categories.

3. Consideration of unequal subgroup sizes for beauty-content exposure

The categories of beauty-related content exposure are highly unbalanced, with very small numbers of participants in some categories. This may influence the stability and interpretation of the statistical analyses. The authors should clarify whether any approach was used to address this issue (e.g., combining categories or sensitivity analysis). At minimum, this limitation should be discussed more explicitly.

4. Avoidance of causal interpretation

Although some improvements have been made, the manuscript still contains several statements that imply causality, which is not appropriate for a cross-sectional study design. For example, terms such as "increases," "strongest predictor," "drives," and "play a decisive role" should be replaced with more appropriate terms indicating association or relationship. The conclusions and discussion should be revised accordingly.

5. Interpretation of regression model findings

The adjusted R² value of 0.21 indicates that the model explains approximately 21% of the variability in cosmetic surgery tendency. This level of explanatory power may be acceptable for psychosocial research; however, the manuscript should acknowledge more clearly that other unmeasured psychological, social, and environmental factors may contribute substantially to cosmetic surgery attitudes. Therefore, statements emphasizing the identified factors as decisive or comprehensive determinants should be moderated.

6. Statistical reporting and transparency

Although the regression analysis has been improved, the authors should ensure complete reporting of regression parameters, including regression coefficients, confidence intervals, p-values, and standard errors. Clear and consistent reporting will improve reproducibility and interpretation of the findings.

7. Final language revision

The manuscript has improved; however, several grammatical issues remain that affect readability and scientific clarity. A final professional English language editing is recommended before publication. For example, some sentences remain unclear, such as:

• At a fixed level of other predictors, 1–10 units (category 2) higher in watching beauty content related to social media was associated with a 8.02 times higher in inclination to undergo cosmetic surgeries... (Lines 290–294). This sentence should be revised for grammatical accuracy and clearer interpretation.

• This implies that common method bias could have occurred to some extent. (Lines 375–377). This sentence is unclear in context and should be rewritten to better explain the intended meaning.

A careful language revision throughout the manuscript is required to improve readability and overall presentation.

Minor points:

1. Terminology regarding predictors

The manuscript frequently uses the term "predictor" throughout the Results, Discussion, and Conclusion sections. While acceptable in statistical modeling, this terminology may imply prediction or causation. Please consider replacing it with "associated factor" or "independent correlate" where appropriate.

2. Interpretation of self-esteem finding

The discussion of the positive relationship between self-esteem and cosmetic surgery tendency has been improved; however, some statements remain unclear. The authors should avoid phrases such as "reverse direction" and instead directly explain that higher self-esteem scores were statistically associated with higher cosmetic surgery tendency in this sample.

**********

what does this mean?). If published, this will include your full peer review and any attached files.

If you choose “no”, your identity will remain anonymous but your review may still be made public.

**Do you want your identity to be public for this peer review?**  For information about this choice, including consent withdrawal, please see our Privacy Policy

Reviewer #1: **Yes:**  Humayun Nadeem

Reviewer #2: No

**********

[NOTE: If reviewer comments were submitted as an attachment file, they will be attached to this email and accessible via the submission site. Please log into your account, locate the manuscript record, and check for the action link "View Attachments". If this link does not appear, there are no attachment files.]

To ensure your figures meet our technical requirements, please review our figure guidelines: https://journals.plos.org/plosone/s/figures

You may also use PLOS’s free figure tool, NAAS, to help you prepare publication quality figures: https://journals.plos.org/plosone/s/figures#loc-tools-for-figure-preparation.

NAAS will assess whether your figures meet our technical requirements by comparing each figure against our figure specifications.

# Author comment: Author response to Decision Letter 2

**DOI:** 10.1371/journal.pone.0360409.r005

19 Aug 2026

Dear reviewers of the manuscript

The authors of the manuscript, once again, say thanks for reviewing this manuscript and providing scientific feedback.

Good Luck.

Attachment Submitted filename: Point-by-Point 14050526+.pdf

# Peer review: Decision Letter 2

**DOI:** 10.1371/journal.pone.0360409.r006

**Authors:** Muhammad Athar

26 Aug 2026

Dear Dr. Ebrahimpour Sadagheyani,

Thank you for submitting your manuscript to PLOS One. After careful consideration, we feel that it has merit but does not fully meet PLOS One’s publication criteria as it currently stands. Therefore, we invite you to submit a revised version of the manuscript that addresses the points raised during the review process.

Please submit your revised manuscript by Sep 25 2026 11:59PM. If you will need more time than this to complete your revisions, please reply to this message or contact the journal office at plosone@plos.org. When you're ready to submit your revision, log on to https://www.editorialmanager.com/pone/ and select the 'Submissions Needing Revision' folder to locate your manuscript file.

- A letter that responds to each point raised by the academic editor and reviewer(s). You should upload this letter as a separate file labeled 'Response to Reviewers'.
- A marked-up copy of your manuscript that highlights changes made to the original version. You should upload this as a separate file labeled 'Revised Manuscript with Track Changes'.
- An unmarked version of your revised paper without tracked changes. You should upload this as a separate file labeled 'Manuscript'.

If you would like to make changes to your financial disclosure, please include your updated statement in your cover letter. Guidelines for resubmitting your figure files are available below the reviewer comments at the end of this letter.

If applicable, we recommend that you deposit your laboratory protocols in protocols.io to enhance the reproducibility of your results. Protocols.io assigns your protocol its own identifier (DOI) so that it can be cited independently in the future. For instructions see: https://journals.plos.org/plosone/s/submission-guidelines#loc-laboratory-protocols. Additionally, PLOS One offers an option for publishing peer-reviewed Lab Protocol articles, which describe protocols hosted on protocols.io. Read more information on sharing protocols at https://plos.org/protocols?utm_medium=editorial-email&utm_source=authorletters&utm_campaign=protocols.

As the corresponding author, your ORCID iD is verified in the submission system and will appear in the published article. PLOS supports the use of ORCID, and we encourage all coauthors to register for an ORCID iD and use it as well. Please encourage your coauthors to verify their ORCID iD within the submission system before final acceptance, as unverified ORCID iDs will not appear in the published article. *Only*  the individual author can complete the verification step; PLOS staff *cannot*  verify ORCID iDs on behalf of authors.

We look forward to receiving your revised manuscript.

Kind regards,

Muhammad Athar, PhD

Academic Editor

PLOS One

Journal Requirements:

If the reviewer comments include a recommendation to cite specific previously published works, please review and evaluate these publications to determine whether they are relevant and should be cited. There is no requirement to cite these works unless the editor has indicated otherwise.

Please review your reference list to ensure that it is complete and correct. If you have cited papers that have been retracted, please include the rationale for doing so in the manuscript text, or remove these references and replace them with relevant current references. Any changes to the reference list should be mentioned in the rebuttal letter that accompanies your revised manuscript. If you need to cite a retracted article, indicate the article’s retracted status in the References list and also include a citation and full reference for the retraction notice.

[Note: HTML markup is below. Please do not edit.]

Reviewers' comments:

Reviewer's Responses to Questions

**Comments to the Author**

Reviewer #2: (No Response)

**********

2. Is the manuscript technically sound, and do the data support the conclusions?

Reviewer #2: Partly

**********

3. Has the statistical analysis been performed appropriately and rigorously?

Reviewer #2: (No Response)

**********

4. Have the authors made all data underlying the findings in their manuscript fully available?

The PLOS Data policy

Reviewer #2: (No Response)

**********

5. Is the manuscript presented in an intelligible fashion and written in standard English?

Reviewer #2: (No Response)

**********

Reviewer #2: The authors have addressed several of the concerns raised in the second round of review, and the manuscript has improved accordingly. However, some issues remain only partially addressed, particularly regarding the coding and interpretation of the beauty-related content exposure variable, the potential effect of unequal subgroup sizes, the continued use of causal or overly strong language in a cross-sectional study, and the completeness of statistical reporting. Following the correction of the Rosenberg Self-Esteem Scale scoring, the authors should also ensure that the revised direction and interpretation of the self-esteem findings are fully consistent throughout the Abstract, Results, Discussion, Conclusion, tables, and supplementary material. Some grammatical and stylistic issues also remain and require final English language editing.

**********

what does this mean?). If published, this will include your full peer review and any attached files.

If you choose “no”, your identity will remain anonymous but your review may still be made public.

**Do you want your identity to be public for this peer review?**  For information about this choice, including consent withdrawal, please see our Privacy Policy

Reviewer #2: No

**********

[NOTE: If reviewer comments were submitted as an attachment file, they will be attached to this email and accessible via the submission site. Please log into your account, locate the manuscript record, and check for the action link "View Attachments". If this link does not appear, there are no attachment files.]

To ensure your figures meet our technical requirements, please review our figure guidelines: https://journals.plos.org/plosone/s/figures

You may also use PLOS’s free figure tool, NAAS, to help you prepare publication quality figures: https://journals.plos.org/plosone/s/figures#loc-tools-for-figure-preparation.

NAAS will assess whether your figures meet our technical requirements by comparing each figure against our figure specifications.

# Author comment: Author response to Decision Letter 3

**DOI:** 10.1371/journal.pone.0360409.r007

30 Aug 2026

Dear Editor-in-Chief; Dear Reviewer

Thank you for reviewing this manuscript in deep. The last uploaded manuscript, compared to the initial manuscript, has more than 90% of its content corrected. Perhaps, like me, you believe that the published manuscript contained some errors. The authors of this manuscript tried to correct errors as well as possible. But some errors can not be corrected. Response to comments:

1- Coding and interpretation of the beauty-related content exposure variable:

Thanks. Tendency toward cosmetic surgery is scored based on questionnaire items (24-120). The cut-off of 72 is the median of the scale scores (24 + 120/2 = 72). Participants with scores more than the median are susceptible to cosmetic surgery. This point was added to the scale explanation in the manuscript (Lines 158-62).

2- The potential effect of unequal subgroup sizes:

Thanks. The unequal sample sizes by gender of students are explained in lines 284-89. This section reviewed similar studies with the same gender ratio.

Dear reviewer: All of the authors of this manuscript are teachers at Khoy University of Medical Sciences and are informed about the gender ratio of males to females in other medical universities. This gender ratio is real in the studied population, especially among medical students. It's a fact that the male-to-female student ratio in some medical courses in Iran (especially Nursing, Public Health, Environmental Health, Laboratory sciences, …) is less than 10%.

3- The continued use of causal or overly strong language in a cross-sectional study, and the completeness of statistical reporting:

Thanks. The authors have tried to prevent causal interpretation of results. Another time, interpretations are checked.

4- Following the correction of the Rosenberg Self-Esteem Scale scoring, the authors should also ensure that the revised direction and interpretation of the self-esteem findings are fully consistent throughout the Abstract, Results, Discussion, Conclusion, tables, and supplementary material.

Thanks. Done.

5- Some grammatical and stylistic issues also remain and require final English language editing.

Thanks. Grammatical errors will be checked again.

Good Luck.

Attachment Submitted filename: Response to Reviewers.docx

# Peer review: Decision Letter 3

**DOI:** 10.1371/journal.pone.0360409.r008

**Authors:** Muhammad Athar

3 Sep 2026

Dear Dr. Ebrahimpour Sadagheyani,

plosone@plos.org. When you're ready to submit your revision, log on to https://www.editorialmanager.com/pone/ and select the 'Submissions Needing Revision' folder to locate your manuscript file.

- A letter that responds to each point raised by the academic editor and reviewer(s). You should upload this letter as a separate file labeled 'Response to Reviewers'.
- A marked-up copy of your manuscript that highlights changes made to the original version. You should upload this as a separate file labeled 'Revised Manuscript with Track Changes'.
- An unmarked version of your revised paper without tracked changes. You should upload this as a separate file labeled 'Manuscript'.

If you would like to make changes to your financial disclosure, please include your updated statement in your cover letter. Guidelines for resubmitting your figure files are available below the reviewer comments at the end of this letter.

If applicable, we recommend that you deposit your laboratory protocols in protocols.io to enhance the reproducibility of your results. Protocols.io assigns your protocol its own identifier (DOI) so that it can be cited independently in the future. For instructions see: https://journals.plos.org/plosone/s/submission-guidelines#loc-laboratory-protocols. Additionally, PLOS One offers an option for publishing peer-reviewed Lab Protocol articles, which describe protocols hosted on protocols.io. Read more information on sharing protocols at https://plos.org/protocols?utm_medium=editorial-email&utm_source=authorletters&utm_campaign=protocols.

As the corresponding author, your ORCID iD is verified in the submission system and will appear in the published article. PLOS supports the use of ORCID, and we encourage all coauthors to register for an ORCID iD and use it as well. Please encourage your coauthors to verify their ORCID iD within the submission system before final acceptance, as unverified ORCID iDs will not appear in the published article. *Only*  the individual author can complete the verification step; PLOS staff *cannot*  verify ORCID iDs on behalf of authors.

We look forward to receiving your revised manuscript.

Kind regards,

Muhammad Athar, PhD

Academic Editor

PLOS One

Journal Requirements:

If the reviewer comments include a recommendation to cite specific previously published works, please review and evaluate these publications to determine whether they are relevant and should be cited. There is no requirement to cite these works unless the editor has indicated otherwise.

Please review your reference list to ensure that it is complete and correct. If you have cited papers that have been retracted, please include the rationale for doing so in the manuscript text, or remove these references and replace them with relevant current references. Any changes to the reference list should be mentioned in the rebuttal letter that accompanies your revised manuscript. If you need to cite a retracted article, indicate the article’s retracted status in the References list and also include a citation and full reference for the retraction notice.

[Note: HTML markup is below. Please do not edit.]

Reviewers' comments:

Reviewer's Responses to Questions

**Comments to the Author**

Reviewer #2: (No Response)

**********

2. Is the manuscript technically sound, and do the data support the conclusions?

Reviewer #2: (No Response)

**********

3. Has the statistical analysis been performed appropriately and rigorously?

Reviewer #2: (No Response)

**********

4. Have the authors made all data underlying the findings in their manuscript fully available?

The PLOS Data policy

Reviewer #2: (No Response)

**********

5. Is the manuscript presented in an intelligible fashion and written in standard English?

Reviewer #2: (No Response)

**********

Reviewer #2: The authors have addressed several concerns raised in the previous round, and the manuscript has improved. However, the following issues still require clarification or correction before publication:

1. Please clarify whether the three exposure categories were entered into the regression using dummy/indicator coding or ordinal coding. The reported coefficient of 7.78 should also be interpreted appropriately for multiple linear regression rather than as “7.78 times more likely.”

2. The beauty-content exposure categories remain highly unbalanced, particularly the highest-exposure group, which contains only a small number of participants. Please discuss how this imbalance may affect the stability and reliability of the regression estimates and the interpretation of this association. If no sensitivity analysis was performed, this should at least be acknowledged as a limitation.

3. The corrected analysis reports an unstandardized coefficient of −1.39 for self-esteem (95% CI: −1.86 to −0.92, P<0.001), indicating an inverse association with cosmetic surgery tendency, while the manuscript says: "self-esteem showed the strongest association (standardized regression coefficient = 0.40)" and then says media watching was 0.31. Please ensure that this revised result is consistently reflected in the Abstract, Results, Discussion, Conclusion, tables, and supplementary material, and that any values or interpretations from the previous analysis have been removed. The authors should also verify that statements regarding the relative strength of this association are based on the corrected standardized coefficients.

4. Standard errors are still not reported in the regression table. Please include them either in the main table or supplementary material, and verify that all reported regression coefficients and interpretations correspond to the corrected analysis.

5. Some causal or overly strong expressions remain, such as “strong factor,” “increasing interest,” and “strongest association.” These should be revised to reflect associations only, given the cross-sectional design. Several grammatical and stylistic problems remain in the revised manuscript, including duplicated wording. A final professional English-language edit is recommended before publication.

Minor point:

The term “predictor” and expressions such as “reverse direction” should be used cautiously or replaced with “associated factor” and “inverse association,” respectively, where appropriate.

**********

what does this mean?). If published, this will include your full peer review and any attached files.

If you choose “no”, your identity will remain anonymous but your review may still be made public.

**Do you want your identity to be public for this peer review?**  For information about this choice, including consent withdrawal, please see our Privacy Policy

Reviewer #2: No

**********

[NOTE: If reviewer comments were submitted as an attachment file, they will be attached to this email and accessible via the submission site. Please log into your account, locate the manuscript record, and check for the action link "View Attachments". If this link does not appear, there are no attachment files.]

To ensure your figures meet our technical requirements, please review our figure guidelines: https://journals.plos.org/plosone/s/figures

You may also use PLOS’s free figure tool, NAAS, to help you prepare publication quality figures: https://journals.plos.org/plosone/s/figures#loc-tools-for-figure-preparation.

NAAS will assess whether your figures meet our technical requirements by comparing each figure against our figure specifications.

# Author comment: Author response to Decision Letter 4

**DOI:** 10.1371/journal.pone.0360409.r009

16 Sep 2026

Thanks a lot for any comments. All of comments surveyed and necessary corrections has been done.

Attachment Submitted filename: Response to Reviewers 14050625.docx

# Peer review: Decision Letter 4

**DOI:** 10.1371/journal.pone.0360409.r010

**Authors:** Muhammad Athar

28 Sep 2026

A Cross-Sectional Study of Cosmetic Surgery Tendency and Its Psychological and Media-Related Factors among Medical Students in 2024-2025

PONE-D-26-20687R4

Dear Dr. Ebrahimpour Sadagheyani,

We’re pleased to inform you that your manuscript has been judged scientifically suitable for publication and will be formally accepted for publication once it meets all outstanding technical requirements.

Within one week, you’ll receive an e-mail detailing the required amendments. When these have been addressed, you’ll receive a formal acceptance letter and your manuscript will be scheduled for publication.

An invoice will be generated when your article is formally accepted. Please note, if your institution has a publishing partnership with PLOS and your article meets the relevant criteria, all or part of your publication costs will be covered. Please make sure your user information is up-to-date by logging into Editorial Manager at Editorial Manager® and clicking the ‘Update My Information' link at the top of the page. For questions related to billing, please contact billing support.

If your institution or institutions have a press office, please notify them about your upcoming paper to help maximize its impact. If they’ll be preparing press materials, please inform our press team as soon as possible -- no later than 48 hours after receiving the formal acceptance. Your manuscript will remain under strict press embargo until 2 pm Eastern Time on the date of publication. For more information, please contact onepress@plos.org.

Kind regards,

Muhammad Athar, PhD

Academic Editor

PLOS One

Additional Editor Comments (optional):

Reviewers' comments:

Reviewer's Responses to Questions

**Comments to the Author**

Reviewer #1: All comments have been addressed

**********

2. Is the manuscript technically sound, and do the data support the conclusions?

Reviewer #1: Yes

**********

3. Has the statistical analysis been performed appropriately and rigorously?

Reviewer #1: Yes

**********

4. Have the authors made all data underlying the findings in their manuscript fully available?

The PLOS Data policy

Reviewer #1: Yes

**********

5. Is the manuscript presented in an intelligible fashion and written in standard English?

Reviewer #1: Yes

**********

Reviewer #1: The authors have revised all the concerns raised previously. I recommend publishing of this manuscript.

**********

what does this mean?). If published, this will include your full peer review and any attached files.

If you choose “no”, your identity will remain anonymous but your review may still be made public.

**Do you want your identity to be public for this peer review?**  For information about this choice, including consent withdrawal, please see our Privacy Policy

Reviewer #1: **Yes:**  Humayun Nadeem

**********

# Editor report: Acceptance letter

**DOI:** 10.1371/journal.pone.0360409.r011

**Authors:** Muhammad Athar

PONE-D-26-20687R4

PLOS One

Dear Dr. Ebrahimpour Sadagheyani,

I'm pleased to inform you that your manuscript has been deemed suitable for publication in PLOS One. Congratulations! Your manuscript is now being handed over to our production team.

At this stage, our production department will prepare your paper for publication. This includes ensuring the following:

* All references, tables, and figures are properly cited

* All relevant supporting information is included in the manuscript submission,

* There are no issues that prevent the paper from being properly typeset

You will receive further instructions from the production team, including instructions on how to review your proof when it is ready. Please keep in mind that we are working through a large volume of accepted articles, so please give us a few days to review your paper and let you know the next and final steps.

Lastly, if your institution or institutions have a press office, please let them know about your upcoming paper now to help maximize its impact. If they'll be preparing press materials, please inform our press team within the next 48 hours. Your manuscript will remain under strict press embargo until 2 pm Eastern Time on the date of publication. For more information, please contact onepress@plos.org.

You will receive an invoice from PLOS for your publication fee after your manuscript has reached the completed accept phase. If you receive an email requesting payment before acceptance or for any other service, this may be a phishing scheme. Learn how to identify phishing emails and protect your accounts at https://explore.plos.org/phishing.

If we can help with anything else, please email us at customercare@plos.org.

Thank you for submitting your work to PLOS One and supporting open access.

Kind regards,

PLOS One Editorial Office Staff

on behalf of

Dr. Muhammad Athar

Academic Editor

PLOS One
